# Circular *PVT1* promotes cardiac fibroblast activation interacting with miR-30a-5p and miR-125b-5p

**DOI:** 10.1038/s41419-025-07652-7

**Published:** 2025-04-21

**Authors:** Alessia Bibi, Alisia Madè, Simona Greco, Jose Manuel Garcia-Manteiga, Anna Sofia Tascini, Spyros Tastsoglou, Germana Zaccagnini, Przemyslaw Leszek, Carlo Gaetano, Fabio Martelli

**Affiliations:** 1 Molecular Cardiology Laboratory, IRCCS Policlinico San Donato, San Donato Milanese, Milan, Italy; 2 Department of Biosciences, University of Milan, Milan, Italy; 3 Center for Omics Sciences, IRCCS Ospedale San Raffaele, Milan, Italy; 4 Università Vita-Salute San Raffaele, Milan, Italy; 5 Department of Heart Failure and Transplantology, Department of Mechanical Circulatory Support and Transplant, National Institute of Cardiology, Warsaw, Poland; 6 Laboratory of Epigenetics, Istituti Clinici Scientifici Maugeri IRCCS, Pavia, Italy; 7 Laboratory of Stem Cell Biology, Institute of Cellular Biology and Pathology “Nicolae Simionescu”, Bucharest, Romania

**Keywords:** Non-coding RNAs, Cardiovascular diseases

## Abstract

Circular RNAs (circRNAs) are involved in the pathogenesis of several cardiovascular diseases, including heart failure. In this study, we report that circular *PVT1* (*circPVT1*) was upregulated in the left ventricle of 31 ischemic heart failure patients compared to 11 non-ischemic controls. RNA sequencing analysis following *circPVT1* knockdown in immortalized human cardiomyocytes identified differentially expressed genes, mainly involved in fibrosis. Notably, in human cardiac fibroblasts, *circPVT1* expression significantly increased after TGF-β1 treatment and *circPVT1* silencing attenuated the levels of pro-fibrotic markers induced by TGF-β1. RNA pull-down assays validated the interaction between *circPVT1* and two fibrosis-related miRNAs, miR-30a-5p and miR-125b-5p. The levels of these miRNAs were not altered upon *circPVT1* knockdown. However, the expression of their mRNA targets was deregulated upon *circPVT1* silencing, suggesting that *circPVT1* modulates miRNA cellular bioavailability. Accordingly, inhibition of either miR-30a-5p or miR-125b-5p restored the expression of TGF-β1-induced pro-fibrotic markers following *circPVT1* silencing, indicating that both miR-30a-5p and miR-125b-5p act as downstream effectors of *circPVT1* in cardiac fibroblast activation. In conclusion, these findings highlight a pro-fibrotic role for *circPVT1*, which can regulate cardiac fibroblast activation interacting with the anti-fibrotic miR-30a-5p and miR-125b-5p. The modulation of *circPVT1* expression may represent a potential strategy to reduce cardiac fibrosis and remodeling.

## Introduction

Circular RNAs (circRNAs) are a re-discovered class of non-coding RNAs originating from the splicing and circularization of precursor mRNAs and long non-coding RNAs [1, 2]. They were first considered as by-products of abnormal splicing with limited functional potential. However, evidence from the ever-increasing high-throughput transcriptome sequencing has identified circRNAs as stable and abundant transcripts in different cell lines and tissues [3, 4]. The functions of circRNAs correlate with their cellular localization. For example, exonic circRNAs are primarily localized within the cytoplasm where they can operate as competing endogenous RNAs (ceRNAs), interacting with miRNAs, thus influencing miRNA specific mRNA targets’ levels. circRNAs can also interact with RNA-binding proteins or be translated into proteins [2, 5–7].

Like other noncoding RNA species, circRNAs have been found to be deregulated in several cardiovascular diseases, including heart failure (HF) [8–10], a leading cause of human morbidity and mortality worldwide [11, 12]. Ischemic cardiomyopathy is characterized by the heart’s decreased ability to pump blood efficiently due to myocardial damage caused by ischemia and is the most common cause of HF in developed countries. Despite advances in the management of HF, it remains a growing public health burden in the elderly population [13].

In a previous study, we identified *circPVT1* as one of the top upregulated circRNAs in ischemic HF (IHF) [14]. *circPVT1(2)* [15] (annotated in circBase as hsa_circ_0001821) is a monoexonic circRNA that is formed by the exon 2 reverse-splicing of the long non-coding RNA originating from the *PVT1 oncogene* (*PVT1*), located on chromosome 8 [16]. In this study, we investigated the transcriptomic changes induced by *circPVT1* silencing and identified its pro-fibrotic role mediated by the interaction with miR-30a-5p and miR-125b-5p.

## Results

### Molecular characterization of cardiac *circPVT1*

**Fig. 1:**
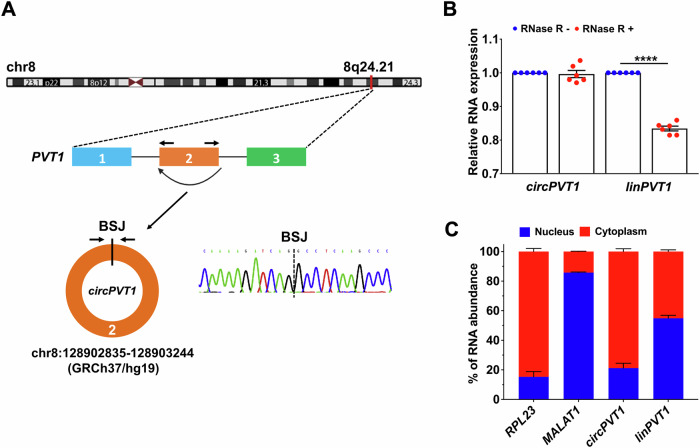
*circPVT1* is a circular RNA mainly located in the cytoplasm. **A** Schematic representation of *PVT1* and *circPVT1* genomic locus and structure. *circPVT1* originates from the circularization of the exon 2 of the transcript originating from the *PVT1* gene on chromosome 8q24.21. RT-qPCR and Sanger sequencing verified the *circPVT1* BSJ sequence expressed in the LV of human hearts. Arrows represent divergent primers used to amplify *circPVT1* BSJ. **B** RT-qPCR analysis of *circPVT1* and linear *PVT1* (*linPVT1*) in LV-derived total RNA treated with (RNase R+) or without (RNase R−) RNase R exonuclease. *circPVT1* was resistant to RNase R treatment whereas linear *PVT1* (*linPVT1*) was significantly degraded. The *circPVT1* and linear *PVT1* (*linPVT1*) levels were normalized to the values detected in the RNase R- condition. Mean values and error bars are shown as ±SEM (*n* = 6 independent biological replicates, ****p < 0.0001). **C** RT-qPCR analysis indicates the abundance of *circPVT1* and linear *PVT1* (*linPVT1*) in either the cytoplasm or the nucleus of AC16 cells. *RPL23* and *MALAT1* were used as cytoplasmic and nuclear markers, respectively (*n* = 3 independent biological replicates).

First, the back-splice junction (BSJ) of *circPVT1* was verified to characterize its structure in the human heart. A divergent primer pair was used to amplify, by RT-qPCR, the BSJ of left ventricle (LV)-derived RNAs, followed by Sanger sequencing of the obtained amplicon (Fig. 1A). Then, two additional divergent primer pairs were used to amplify the entire sequence of *circPVT1*. Agarose gel electrophoresis and Sanger sequencing confirmed that *circPVT1* was 410 nucleotides long and encompassed the entire length of exon 2 of *PVT1* (Supplementary Fig. S1). Finally, the resistance against RNase R exonuclease digestion [17] compared to linear *PVT1* confirmed the absence of a 3’-end and the circular structure of *circPVT1* (Fig. 1B).

Most exonic circRNAs are located in the cytoplasm [18]. Nuclear/cytoplasmic cellular fractionation experiments in AC16 cardiomyocyte cell line revealed that *circPVT1* prominently localized in the cytoplasm, while the linear form is distributed at similar levels in the cytoplasmic and in the nuclear fractions (Fig. 1C). Additionally, we evaluated the expression of *circPVT1* in relevant myocardial cell types such as cardiomyocytes, cardiac fibroblasts and endothelial cells. *circPVT1* was readily detectable in all the tested cell types (Supplementary Table S1), indicating its ubiquitous expression in the heart.

In conclusion, *circPVT1* is a circular RNA ubiquitously expressed in the heart and is mainly located in the cytoplasm.

### *circPVT1* is increased in end-stage IHF patients, and in a mouse model of myocardial infarction

We previously found that *circPVT1* was significantly increased in non-end-stage IHF patients and the *circPVT1*-to-linear *PVT1* ratio showed a similar modulation of *circPVT1*, suggesting that the differential expression of *circPVT1* was independent of the transcriptional regulation of its host gene [14]. To further validate the modulation of *circPVT1* upon IHF, we measured the circular and linear *PVT1* levels in the LV of patients with end-stage IHF who underwent heart transplantation. *circPVT1* levels were elevated in end-stage IHF patients, while the levels of linear *PVT1* showed no significant changes (Supplementary Fig. S2). Accordingly, the RNA circular-to-linear ratio was also higher in IHF patients (Supplementary Fig. S2). These findings indicate that the up-modulation of *circPVT1* we had previously observed in non-end-stage IHF patients is sustained until end-stage IHF [14].

Then, we investigated whether *circPVT1* was similarly modulated in a mouse model of ischemic cardiomyopathy. To this aim, an acute myocardial infarction (AMI) model was established by ligating the left anterior descending coronary artery. LV fractional shortening (FS) value, *circPVT1* and linear *PVT1* abundance were analyzed at 7 and 14 days after the procedure. Sham-operated mice were used as controls. Echocardiography indicated that, as expected, FS was significantly lower in the AMI group compared to the controls (Supplementary Fig. S3).

We found that *circPVT1* levels significantly increased at both 7 and 14 days (Supplementary Fig. S4A). Interestingly, linear *PVT1* was also increased upon AMI (Supplementary Fig. S4B). These results suggest that also in mice *circPVT1* is increased in ischemic hearts, although with a different mechanism than in humans.

### Silencing of *circPVT1* modulates fibrosis and senescence-associated genes

We designed siRNAs targeting the BSJ of *circPVT1* to investigate the functional role of *circPVT1*. siRNAs targeting the linear *PVT1* transcript were also generated. Altogether, 6 siRNAs were screened, and the comparison to negative control (i.e., a non-targeting siRNA) allowed the identification of siRNAs that either efficiently knocked down the circular but did not affect the linear isoform of *PVT1* (Supplementary Fig. S5A) or, conversely, that silenced the linear but not the *circPVT1* form (Supplementary Fig. S5B). Therefore, we selected siRNA *cPVT1_1* and siRNA *cPVT1_3* to target *circPVT1* and siRNA *linPVT1_2* to target the host mRNA in the following experiments.

We performed RNA sequencing of AC16 cells transfected with siRNAs targeting *circPVT1* or linear *PVT1* or with a non-targeting siRNA control to assess transcriptomic changes upon *circPVT1* knockdown. We analyzed the differentially expressed genes in *circPVT1* knockdown cells compared to cells transfected with non-targeting siRNA control and compared to linear *PVT1* knockdown samples. To identify genes specifically modulated by *circPVT1* knockdown, only overlapping genes in these two differential expression analyses were considered, identifying 1370 genes in common. Pathway analysis showed that these genes were mainly involved in extracellular matrix (ECM) formation, fibrosis, and cellular senescence (Supplementary Fig. S6), suggesting the involvement of *circPVT1* in cardiac remodeling and fibrosis.

### *circPVT1* knockdown attenuates the expression of pro-fibrotic markers and TGF-β1 signaling

**Fig. 2:**
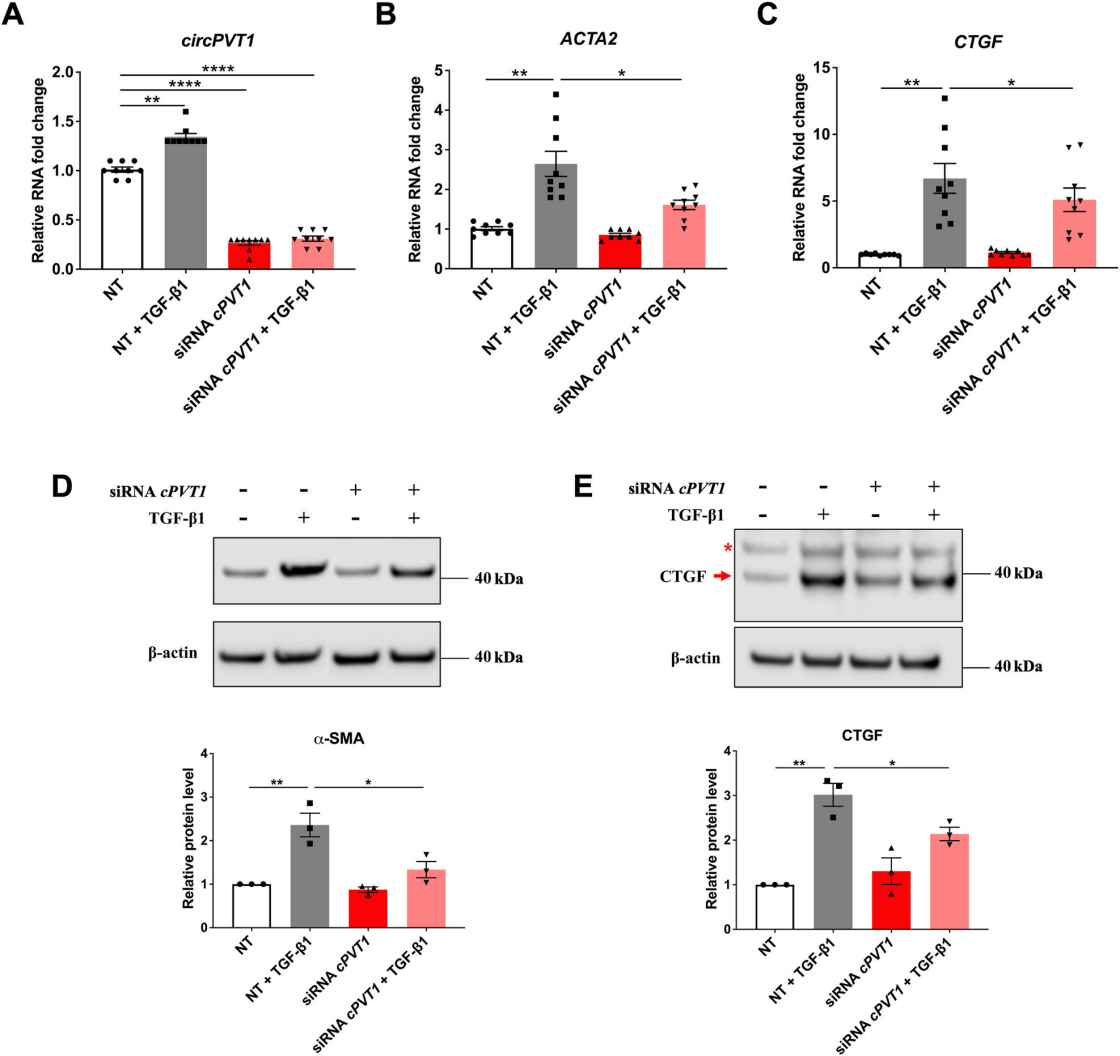
The knockdown of *circPVT1* reduces TGF-β1-induced expression of pro-fibrotic markers. HCF were transfected with non-targeting siRNA (NT) or *circPVT1*_1 siRNA (siRNA *cPVT1*) and then stimulated with TGF-β1 for 24 h. RT-qPCR analysis of *circPVT1* (**A**), *ACTA2* (**B**), and *CTGF* (**C**) expression mean values are shown in fold change ± SEM (*n* = 9 independent biological replicates, *p < 0.05, **p < 0.01, ****p < 0.0001). Representative western blot (top) and quantitative analysis (bottom) of α-SMA (**D**) and CTGF (**E**) protein levels. The red arrow indicates the CTGF band; * indicates a non-specific band. The density of α-SMA and CTGF bands were normalized to that of β-actin; a 11–250 kDa protein ladder was used as molecular weight reference. Data are presented as mean ± SEM from three independent experiments (*n* = 3, *p < 0.05, **p < 0.01).

As the transcriptomics changes induced by *circPVT1* silencing indicated pathways involved in the fibrosis process, the role of *circPVT1* was investigated in cardiac fibroblasts. We first assessed the expression of *circPVT1* and linear *PVT1* in human cardiac fibroblasts (HCF) by performing absolute quantification using digital droplet PCR (ddPCR). Data revealed that *circPVT1* was expressed at levels comparable to those of linear *PVT1* (*circPVT1* = 27.8 ± 1.9 vs. linear *PVT1* = 43.2 ± 2.1 copies/ng of total RNA), in keeping with similar average Ct values in qPCR reactions (Ct *circPVT1*: 26.0 ± 0.1; Ct linear *PVT1*: 25.7 ± 0.2). HCF cells were then transfected with siRNA *cPVT1_1* and treated with TGF-β1, to model cardiac fibrosis in vitro. RT-qPCR data showed that TGF-β1 stimulation significantly increased *circPVT1* expression in HCF transfected with control non-targeting oligonucleotides, indicating a responsiveness of this circRNA to TGF-β1 (Fig. 2A). As expected, the treatment of HCF cells with TGF-β1 increased the expression of the pro-fibrotic markers *ACTA2*/α-SMA and CTGF. However, the increase was attenuated upon *circPVT1* silencing at both mRNA (Fig. 2B, C) and protein levels (Fig. 2D, E). These findings were further confirmed by transfecting HCF with an independent siRNA, *cPVT1_3* (Supplementary Fig. S7A, B). Upon cell exposure to TGF-β1, *circPVT1* silencing attenuated the mRNA levels of *ACTA2* and *CTGF* and of an additional fibrosis marker, *periostin* (*POSTN*, Supplementary Fig. S7C).

**Fig. 3:**
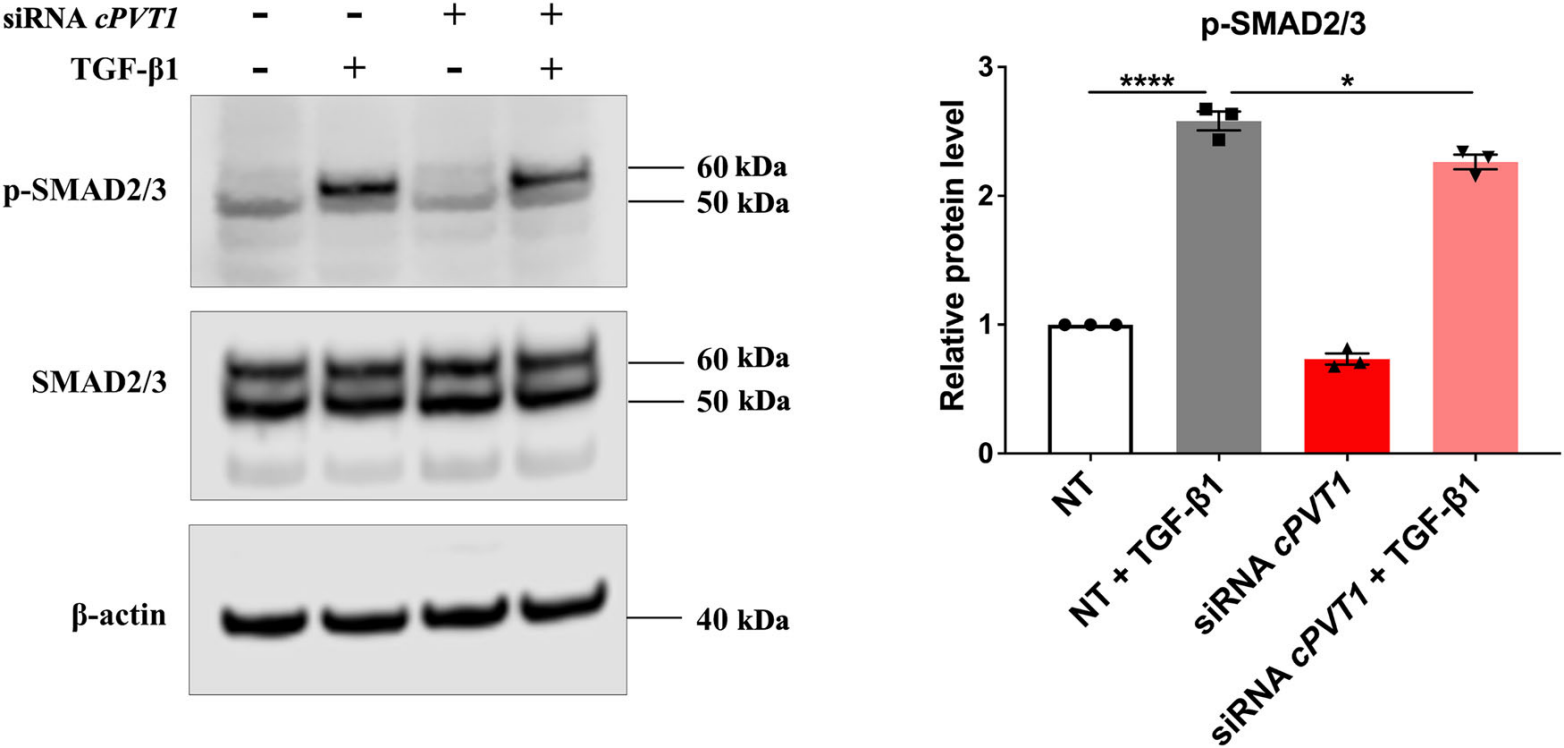
The knockdown of *circPVT1* attenuates TGF-β1-induced SMAD2/3 phosphorylation. HCF cells were transfected with non-targeting siRNA (NT) or *circPVT1*_1 siRNA (siRNA *cPVT1*) and then stimulated with TGF-β1 for 1 h. Western blot (left) and quantitative analysis (right) of p-SMAD2/3 and total SMAD2/3 protein levels. The density of the p-SMAD2/3 band was normalized to that of the total SMAD2/3 protein. 11–250 kDa protein ladder was used as a molecular weight reference. Data are presented as mean ± SEM from three independent experiments (*n* = 3, *p < 0.05, ****p < 0.0001).

TGF-β1-signaling pathways are activated via a phosphorylation cascade involving the SMAD2/3 transcription factor, which drives the expression of crucial pro-fibrotic genes [19, 20]. As expected, in response to TGF-β1 treatment of HCF cells, increased levels of phosphorylated SMAD2/3 protein were observed (Fig. 3). Interestingly, SMAD2/3 phosphorylation was reduced by *circPVT1* knockdown (Fig. 3), indicating that *circPVT1* participates in the TGF-β1-induced fibrosis *via* regulation of the TGF-β1-SMAD pathway.

These data demonstrate that *circPVT1* knockdown limited TGF-β1-induced activation of cardiac fibroblasts, suggesting a pro-fibrotic role of *circPVT1*.

### *circPVT1* interacts with miR-30a-5p and miR-125b-5p

Our previous study on circRNA/miRNA networks in IHF [14] identified miR-30a-5p and miR-30d-5p as predicted interactors of *circPVT1*, both related to fibrosis pathogenetic mechanisms [21–23].

We profiled miRNAs interacting with *circPVT1* following circRNA pull-down in HCF to obtain a more comprehensive picture of *circPVT1*-miRNA interactions, (Supplementary Figure S8A). To this aim, *circPVT1* pull-down was performed in quadruplicate using a biotin-labeled antisense oligo targeting *circPVT1* BSJ (*circPVT1*-ASO), while a non-targeting oligonucleotide (NEG CTRL ASO) was used as a negative control. *circPVT1* was enriched in the *circPVT1*-ASO pull-down, as expected, confirming the efficiency of the biotinylated antisense oligo (Supplementary Figure S8B). RNA sequencing of the co-purified RNAs identified 7 differentially enriched miRNAs (Supplementary Table S2). We focused on miR-125b-5p, a known regulator of fibrosis [24–29].

**Fig. 4:**
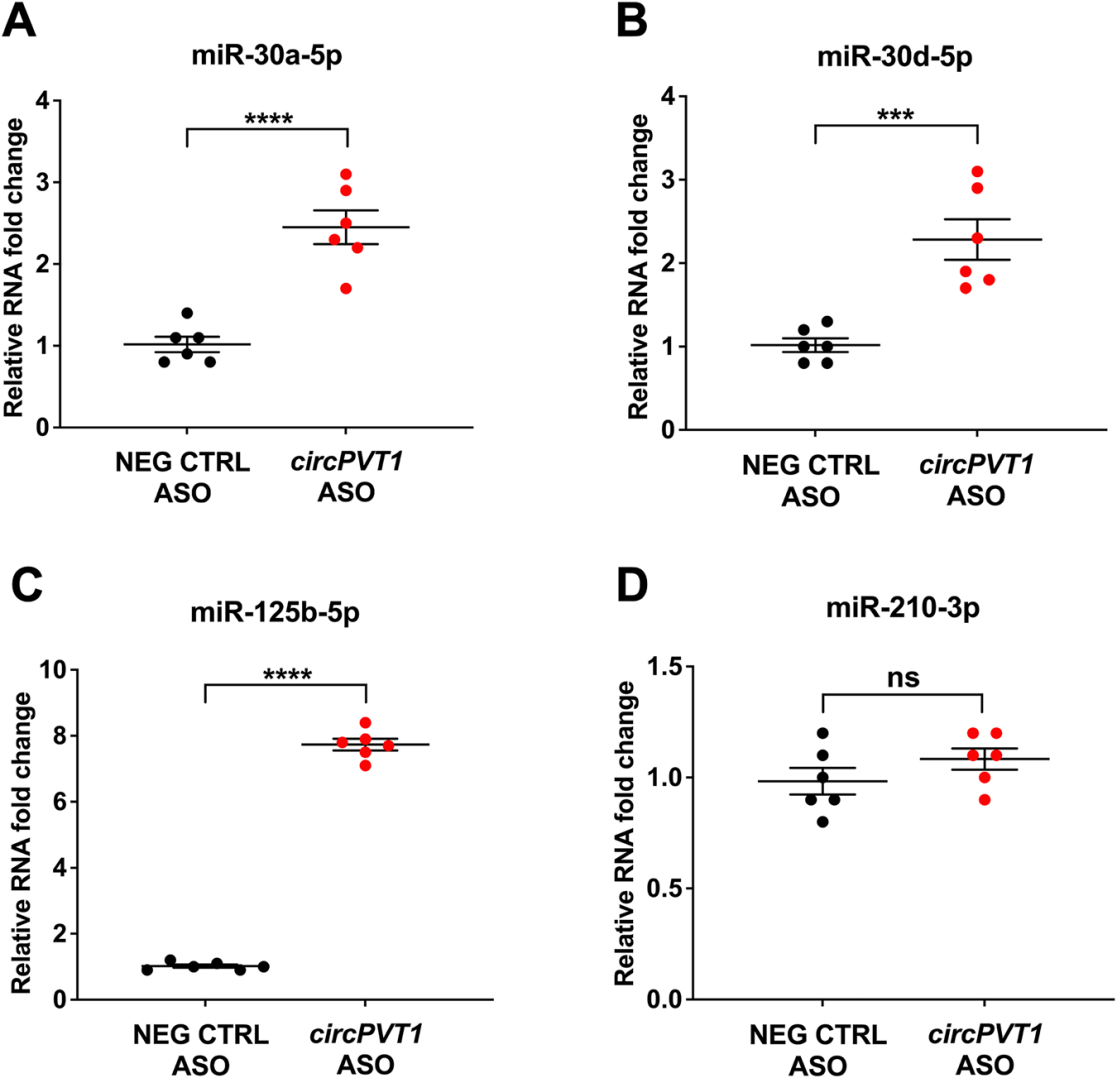
miR-30a-5p, miR-30d-5p and miR-125b-5p enrichment in *circPVT1* pull-downs. Biotinylated ASOs targeting the *circPVT1* BSJ (*circPVT1* ASO) and biotinylated non-targeting control oligonucleotides (NEG CTRL ASO) were used in pull-down experiments against HCF lysates. Relative expression levels of co-purified miR-30a-5p (**A**), miR-30d-5p (**B**), miR-125b-5p (**C**), and miR-210-3p (**D**) were evaluated by RT-qPCR. miR-210-3p, not a *circPVT1* interactor, was used as a negative control. Mean values and error bars are shown in fold change ± SEM (*n* = 6 independent biological replicates, ***p < 0.001, ****p < 0.0001, ns not statistically significant).

The levels of miR-30a/d-5p and miR-125b-5p were measured by RT-qPCR in independent *circPVT1* pull-downs carried out in HCF to validate these miRNA-*circPVT1* interactions. All miRNAs were enriched in *circPVT1*-ASO RNA pull-downs (Fig. 4A–C). Conversely, miR-210-3p, which was not a predicted *circPVT1* interactor, did not display any enrichment, confirming the specificity of the assay (Fig. 4).

**Fig. 5:**
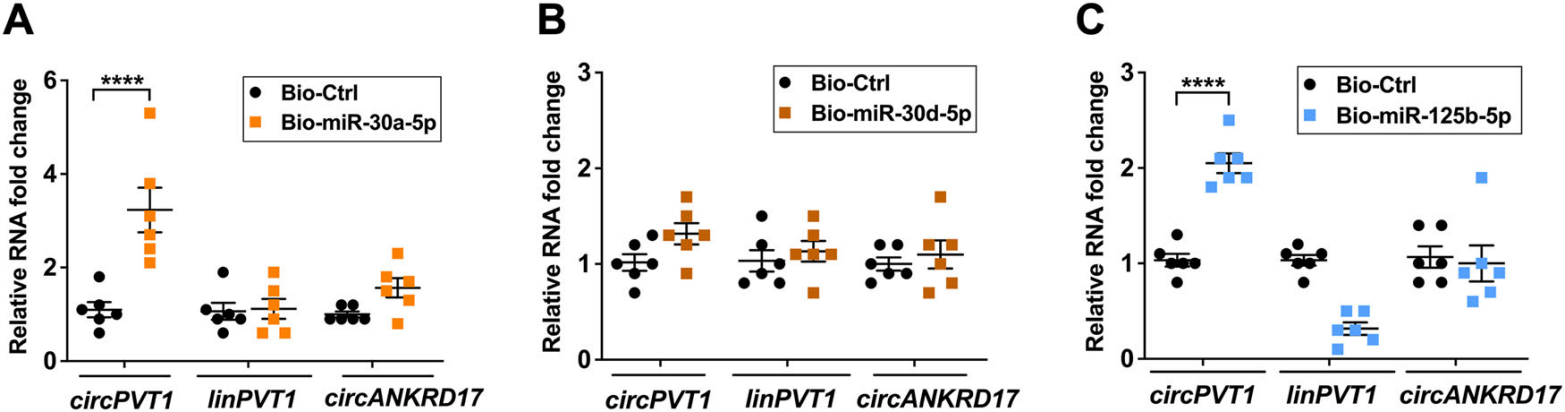
Specific *circPVT1* enrichment in miR-30a-5p and miR-125b-5p pull-downs. Biotinylated miRNAs (bio-miR-30a-5p, bio-miR-30d-5p and bio-miR-125b-5p) or biotinylated miRNA control (bio-Ctrl) were transfected in HCF and 48 h later pulled-down using streptavidin-coupled beads. Relative levels of *circPVT1*, linear *PVT1* or another circRNA, which was not a potential interactor of these miRNAs (*circANKRD17*, negative control) were evaluated by RT-qPCR in miR-30a-5p (**A**), miR-30d-5p (**B**) and miR-125b-5p (**C**) pull-down samples. Mean values are indicated in fold change ± SEM (*n* = 6 independent biological replicates, ****p < 0.0001).

A reverse approach was used to further validate circRNA-miRNA binding, by pulling-down miRNAs and testing their binding to *circPVT1* (Supplementary Fig. S9A). The enrichment of biotinylated miRNAs was first verified in pull-downs, confirming the technique’s efficiency (Supplementary Fig. S9B–D). *circPVT1* was enriched in miR-30a-5p- and miR-125b-5p-captured fractions compared with the non-targeting negative control (Fig. 5A, C), but not in miR-30d-5p pull-down (Fig. 5B). Of note, non-targeted circRNA *circANKRD17* and linear *PVT1* were not significantly enriched, further confirming the assay’s specificity. Thus, we opted to characterize in more detail the interactions of *circPVT1* with miR-30a-5p and miR-125b-5p, while the *circPVT1*/miR-30d-5p pair was not investigated further, as it was not fully validated.

### Secondary structure analysis of *circPVT1* and linear *PVT1*

The accessibility of miR-125b-5p and miR-30a-5p MiRNA Recognition Elements (MREs) on *circPVT1*, also in comparison with its linear counterpart, was assessed by making use of publicly available SHAPE Mutational Profiling (SHAPE-MaP) data [30] and performing SHAPE-MaP-informed secondary structure prediction. Briefly, in SHAPE-MaP, reagents (e.g., 1M7, NMIA) are used to modify RNA and purposefully form adducts at unbound RNA nucleotides. Mutations that are introduced during reverse transcription in these adduct regions are recorded via sequencing to create accessibility profiles, while recordings from corresponding non-modified DMSO samples and denaturing control samples are used to filter and normalize these profiles.

Supplementary Fig. 10 shows that, while certain parts of the exon 2 structure appeared quite similar between the circular and the linear form, linear *PVT1* predicted interplay with the following exon affected the structure in the proximity of the sequences involved in the BSJ (Supplementary Fig. S10A).

Moreover, when the MREs were analyzed, it was found that the region harboring the miR-125b-5p MRE exhibited a more prominently bound-state within the miRNA “seed” region (positions 2 to 8 relative to the miRNA) in the linear prediction, compared to the circular predicted structure, indicating that the *circPVT1* form may exhibit increased accessibility on that site (Supplementary Fig. S10B, D). On the contrary, both forms exhibited similar structure on the region containing the miR-30a-5p site (Supplementary Fig. S10C, D) and, notably, robustly supported the accessibility of the locus binding the miRNA “seed” region.

### Knockdown of *circPVT1* modulates the cellular bioavailability of miR-30a-5p and miR-125b-5p

To investigate the functional interaction between *circPVT1* and miR-30a-5p/miR-125b-5p, we verified whether the expression of these miRNAs was modulated following the silencing of *circPVT1*. RT-qPCR analysis showed that expression levels of miR-30a-5p and miR-125b-5p were not changed upon silencing of *circPVT1*, both in the absence or presence of TGF-β1 (Supplementary Fig. S11A, B). Similarly, *circPVT1* levels were unaffected by miR-30a-5p and miR-125b-5p inhibition, respectively (Supplementary Fig. S11C).

**Fig. 6:**
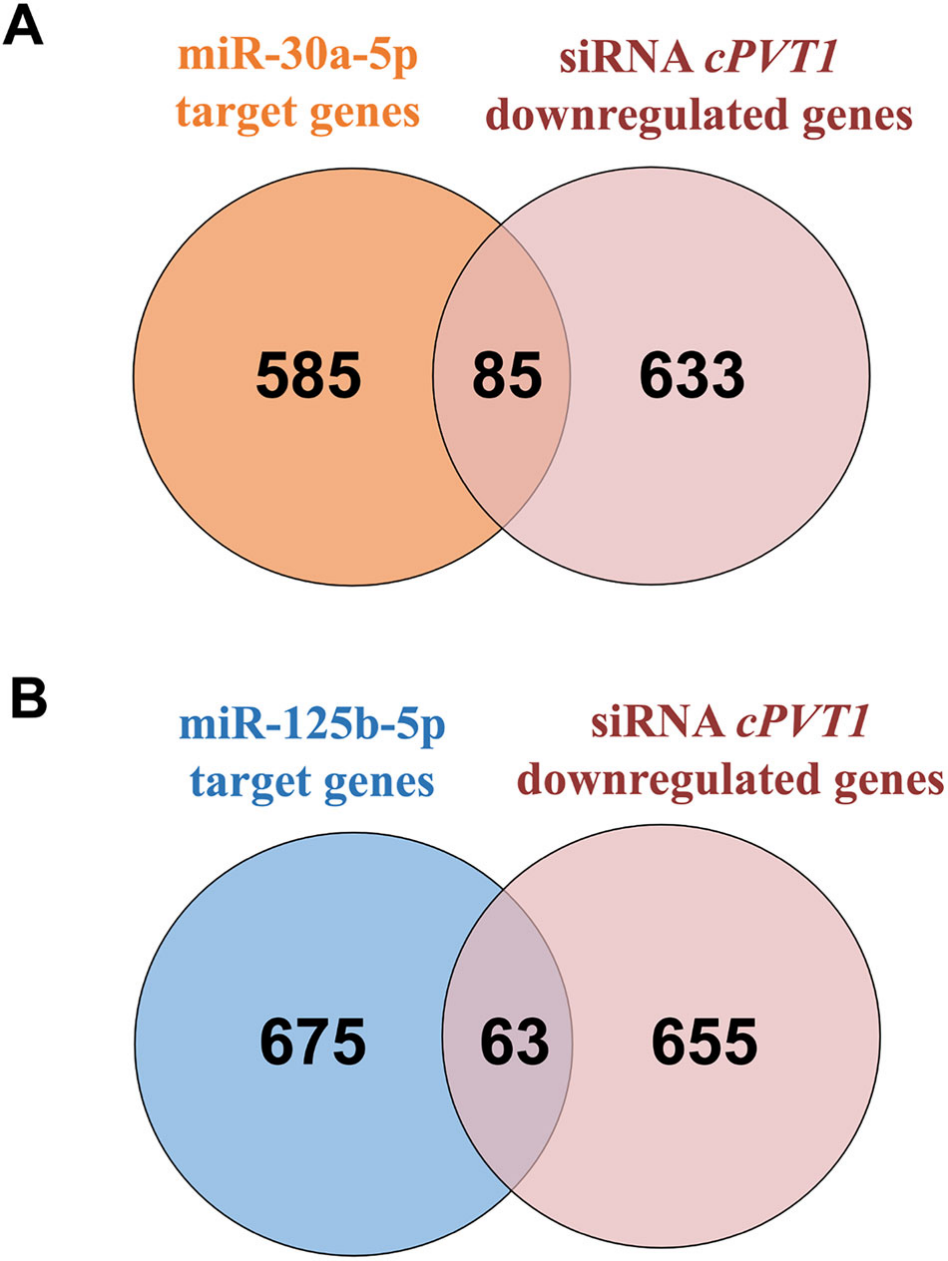
miR-30a-5p and miR-125b-5p predicted targets are deregulated upon *circPVT1* knockdown. Venn diagrams showing statistically significant overlaps between genes significantly downregulated in *circPVT1*-knockdown cells (in light pink) and miR-30a-5p (**A**) and miR-125b-5p (**B**) predicted target genes. The overlap was statistically significant for both miRNAs (*p* < 0.0001).

We next investigated whether *circPVT1*/miRNA interaction led to increased repression of miR-30a-5p and miR-125b-5p targets upon *circPVT1* silencing. To this aim, the mirDIP [31] miRNA target prediction program was interrogated to identify the predicted targets of miR-30a-5p and miR-125b-5p, and these miRNA targets were overlapped with the genes significantly downregulated upon *circPVT1* knockdown, as identified by transcriptomic analysis. The overlap was statistically significant for both miRNAs (Fig. 6), indicating that *circPVT1* can interact with these miRNAs. Indeed, decreased levels of *circPVT1* may de-repress the partner of miRNAs and increase the cellular pool of bioavailable miRNAs, thus enhancing miRNA-mediated repression of the respective target genes.

### miR-30a-5p and miR-125b-5p inhibition restores cardiac fibroblast activation upon *circPVT1* silencing

Literature data showed that miR-30a-5p and miR-125b-5p regulate fibrosis [21, 22, 24–29, 32]. Using the mirDIP database [31], which integrates different prediction tools, *CTGF* and *POSTN* were identified as potential targets of both miR-30a-5p and miR-125b-5p, while the latter miRNA was also predicted to target *ACTA2*. Accordingly, TarBase [33], a database of experimentally supported miRNA/target interactions, also reported *CTGF* and *POSTN* as direct targets of miR-30a-5p.

**Fig. 7:**
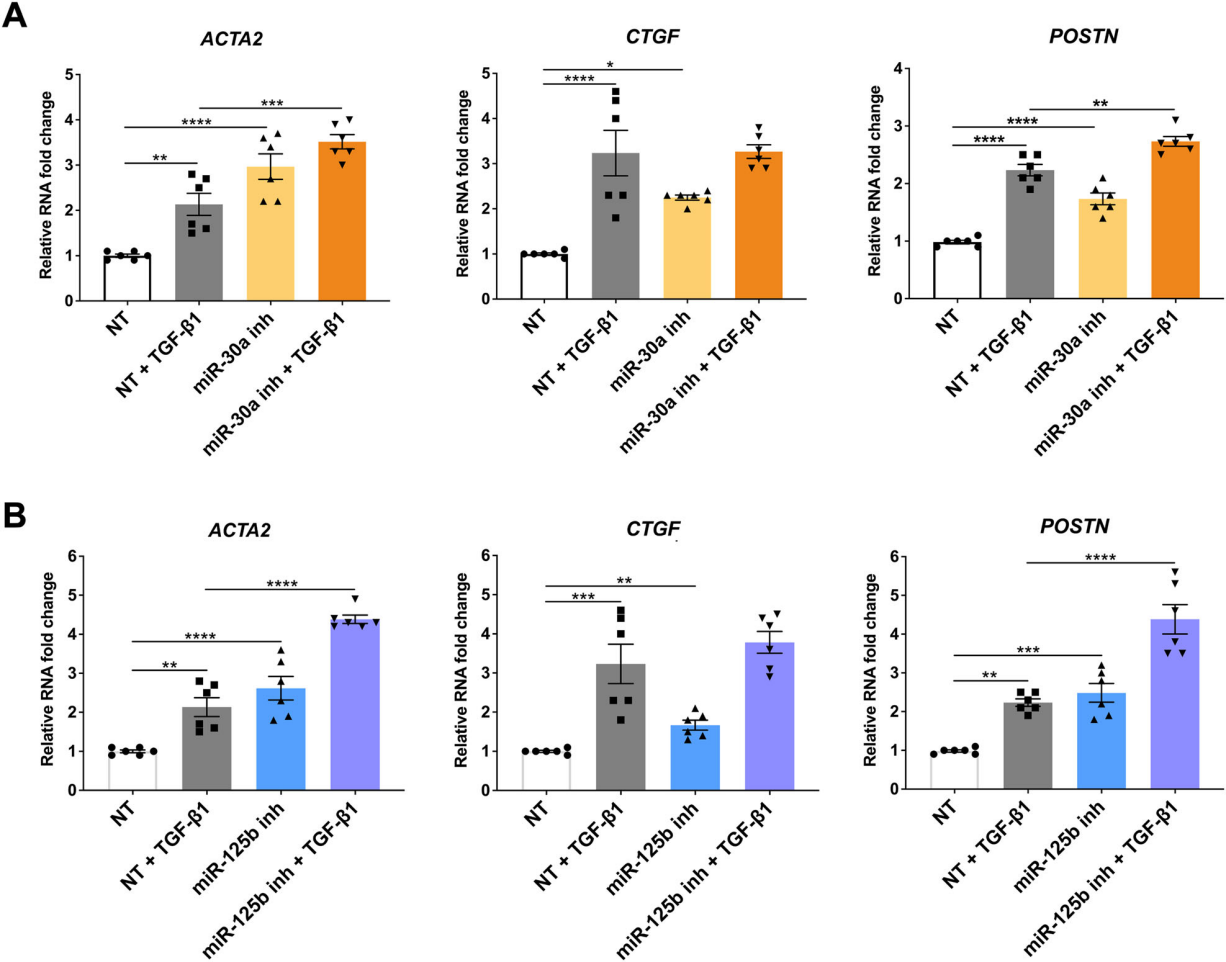
The inhibition of miR-30a-5p or miR-125b-5p increases the expression of pro-fibrotic markers. HCF cells were transfected with miR-30a-5p inhibitor (miR-30a inh) (**A**) or miR-125b-5p inhibitor (miR-125b inh) (**B**) oligonucleotides and then stimulated with TGF-β1 for 24 h. The expression of *ACTA2*, *CTGF* and *POSTN* were measured using RT-qPCR. Data are shown in fold change as mean ± SEM (*n* = 6 independent biological replicates, *p < 0.05, **p < 0.01, ***p < 0.001, ****p < 0.0001).

We transfected HCF with miR-30a-5p and miR-125b-5p inhibitors, before treating the cells with TGF-β1 for 24 h, to confirm these findings and investigate the role of these miRNAs in cardiac fibroblast activation. Figure 7 shows that miR-30a-5p or miR-125b-5p inhibition increased the expression of pro-fibrotic markers *ACTA2*, *CTGF*, and *POSTN*. These results indicate that both miR-30a-5p and miR-125b-5p displayed an anti-fibrotic role in the tested experimental conditions.

**Fig. 8:**
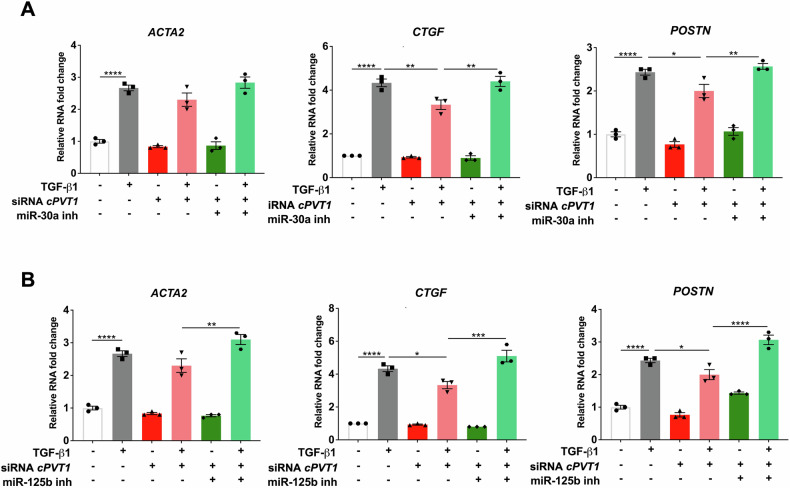
The inhibition of miR-30a-5p or miR-125b-5p rescues cardiac fibroblast activation mediated by *circPVT1* silencing. HCF were transfected with non-targeting siRNA or *circPVT1*_3 siRNA (siRNA *cPVT1*) or *circPVT1*_3 siRNA (siRNA *cPVT1*) + miR-30a-5p inhibitor (miR-30a inh) (**A**), or *circPVT1*_3 siRNA (siRNA *cPVT1*) + miR-125b-5p inhibitor (miR-125b inh) (**B**) and then stimulated with TGF-β1 for 24 h. The expression of *ACTA2*, *CTGF* and *POSTN* were measured using RT-qPCR. Fold change values are presented as mean ± SEM (*n* = 3 independent biological replicates, *p < 0.05, **p < 0.01, ****p < 0.0001).

Then, *circPVT1* siRNA and inhibitors against miR-30a-5p or miR-125b-5p were transfected in HCF cells, before being stimulated with TGF-β1, to elucidate whether the role exerted by *circPVT1* in cardiac fibroblast activation was mediated by miR-30a-5p or miR-125b-5p. We found that the decrease of TGF-β1-induced expression of pro-fibrotic markers *ACTA2*, *CTGF* and *POSTN* in *circPVT1*-knockdown cells was prevented by the concomitant miR-30a-5p (Fig. 8A) or miR-125b-5p (Fig. 8B) inhibition. These findings indicate that miR-125b-5p and miR-30a-5p act as downstream effectors in *circPVT1*-mediated cardiac fibroblast activation.

## Discussion

We previously found that *circPVT1* is a component of a circRNA-centered RNA network in the LV of non-end-stage IHF patients [14], and we further confirmed this finding in end-stage failing hearts. Interestingly, *circPVT1* was one of the top up-regulated circRNAs in IHF, and this was also true when circular-to-linear ratios were analyzed, indicating an independent regulation of the two forms [14]. Accordingly, *circPVT1* is transcribed from a promoter distinct from the linear *PVT1* in head and neck squamous cell carcinoma cells, indicating an independent regulation of their expression [34]. While increased transcription of the specific precursor-RNA giving rise to *circPVT1* may likely explain the observed *circPVT1* increase in IHF, other mechanisms, such as increased circularization efficiency, are also possible. Notably, *PVT1* exon 2, which is back-spliced in *circPVT1*, is preceded and followed in the human genome by two long introns bearing many Alu repeats, likely promoting the biogenesis of *circPVT1* [35].

The *circPVT1* increase upon ischemic cardiomyopathy was also confirmed in a mouse model of AMI, in keeping with previous observations limited to 3 days after the infarction [36]. However, the linear *PVT1* counterpart was also increased in the infarcted heart of the mouse, indicating a difference in the modulation of the *PVT1* gene locus between mice and humans.

Following *circPVT1* knockdown in immortalized human cardiomyocytes, transcriptomics profiling showed that regulation of ECM, fibrosis, and cellular senescence were among the main enriched biological pathways. Panda et al. [16] previously showed that *circPVT1* is a negative regulator of senescence in human fibroblasts, corroborating our findings. However, clinical evidence and experimental studies have shown that senescent cells increase in failing hearts [37]. As *circPVT1* is upregulated in IHF patients [14], while being downregulated in senescent fibroblasts [16], we instead focused on investigating the potential role of *circPVT1* in cardiac fibrosis.

Cardiac fibrosis is characterized by the excessive deposition of ECM proteins, resulting in myocardial stiffness, and is a hallmark of adverse cardiac remodeling in HF [38]. Persistent activation of the TGF-β1 signaling pathway is one of the main factors in inducing myocardial fibrosis [39–41]. The current study showed a significant increase in *circPVT1* expression in HCF after TGF-β1 treatment. Moreover, *circPVT1* knockdown dampened TGF-β1-induced expression of fibrosis markers. In particular, *POSTN* and *CTGF* are two important pro-fibrotic mediators promoting myofibroblast transdifferentiation and ECM production [42–45]. Moreover, *circPVT1* silencing attenuated the expression levels of phosphorylated SMAD2/3 protein, a downstream intracellular effector of TGF-β1 signaling which regulates the expression of crucial pro-fibrotic genes [40, 46]. These findings indicate that *circPVT1* is upregulated in response to TGF-β1 fibrotic stimulus and plays a functional role in cardiac fibroblast activation.

Several studies investigated the role of *circPVT1* in different cancer types, finding that *circPVT1* increases the proliferation, migration, and invasion of tumor cells [47, 48]. While these specific aspects were not investigated in this study, enhanced proliferation and migration are in keeping with a *circPVT1* pro-fibrotic function. Moreover, *circPVT1* promotes epithelial to mesenchymal transition (EMT) in gastric and other cancer types [49–52]. Notably, EMT also plays a role in the genesis of fibroblasts during fibrosis in several organs, including the heart [53–55], corroborating a pro-fibrotic role of *circPVT1*.

Growing evidence indicates that circRNAs can operate as ceRNAs, interacting with miRNAs and modulating the expression of their target genes [5–7, 9]. Indeed, we previously identified miR-30a-5p as a *circPVT1* interactor in a circRNA-centered RNA network characterizing IHF [14]. Pull-down assays confirmed the validity of miR-30a-5p-*circPVT1* interaction and identified the binding of *circPVT1* with miR-125b-5p in HCF. In agreement with our findings, studies in tumor cells provide evidence of the interaction between *circPVT1* and both miR-30a-5p and miR-125b-5p [56–58]. Moreover, *circPVT1* interacts with miR-125b-5p in mouse cardiomyocytes too [36].

miR-30a-5p and miR-125b-5p pull-down experiments indicated a preferential binding of these miRNAs to *circPVT1 vs* its linear counterpart, although the same MREs are present in both *PVT1* RNA isoforms. This data is in line with the SHAPE-MaP-informed RNA folding structures of the exon 2 sequence in its circular and linear forms that we obtained by bioinformatics. The analysis indicated differences in the structure/accessibility of the miR-125b-5p miRNA-recognition element. Moreover, other sequences in the proximity of the circRNA BSJ were predicted to have different structural features than the linear counterpart, possibly generating or disrupting binding sites for RNA-binding proteins that may further affect RNA folding. A limitation of this analysis is the fact that the circ/linear *PVT1* SHAPE-MaP experiments were conducted in ovarian cancer PA-1 cells, which could introduce context-specific bias in the produced RNA structures compared to cardiac cells.

We did not identify previously described *circPVT1*-interacting miRNAs in *circPVT1* pull-downs followed by RNA sequencing performed in HCF, including let-7 [16, 59], most likely due to differences in the adopted experimental systems and the highly stringent selection criteria.

Expression levels of miR-30a-5p and miR-125b-5p did not change upon silencing of *circPVT1*, either in the absence or presence of TGF-β1, as circRNA/miRNA interaction does not always lead to the decrease of the miRNA levels [60–62]. However, miR-30a-5p and miR-125b-5p predictive targets were deregulated upon *circPVT1* silencing, indicating that decreased levels of *circPVT1* could increase the miRNA bioavailability in the cells. This, in turn, results in the reduction of miRNA target levels or in the inhibition of mRNA translation.

Multiple mechanisms may account for the regulation of miR-30a-5p and miR-125b-5p function by *circPVT1*. Considering that only one MRE has been identified for both miR-30a-5p and miR-125b-5p, one possibility is that *circPVT1* operates through sub-stoichiometric interactions, acting as a molecular platform that facilitates miRNA clustering or co-regulation in specific subcellular compartments, as it has been reported for some circRNAs [63–65]. In this scenario, *circPVT1* may enhance the local concentration of miRNAs, increasing their availability for interactions with target mRNAs and modulating fibrotic pathways.

In line with this hypothesis, functional analysis showed that the reduced expression of pro-fibrotic markers, resulting from *circPVT1* silencing, was significantly counteracted by inhibitors of either miR-30a-5p or miR-125b-5p, indicating that both miR-30a-5p and miR-125b-5p act as downstream effectors in *circPVT1*-mediated HCF activation. Indeed, both miRNAs share a significant number of target genes, including critical fibrosis drivers, such as *POSTN* and *CTGF*. In line with our data, direct targeting by miR-30a-5p of *CTGF* and *POSTN* was described in other experimental systems [21, 66].

These results are in agreement with previous studies indicating that miR-30a-5p is a negative regulator of myocardial fibrosis [21, 22, 66]. There are conflicting results about the role of miR-125b-5p role in cardiac fibrosis. Some studies show that miR-125b-5p promotes cardiac fibrosis by inducing proliferation and activation of cardiac fibroblasts [27, 28], but an anti-fibrotic role has also been reported [24, 25, 29]. These apparent discrepancies may be explained by considering the different cell types and physio-pathological contexts that could result in distinct sets of miRNA targets and respective signaling pathways being repressed in each case. Indeed, single-cell transcriptomics characterization of HCF indicates that they are constituted by a pool of functionally distinct cell populations [67–69]. Further research is needed to better understand the complex role of miR-125b-5p in cardiac fibrosis.

Of note, *circPVT1* site-directed mutagenesis and rescue experiments were not performed to further validate *circPVT1* and miR-30a-5p/miR-125b-5p interactions in cardiac fibroblasts. This limitation arose from the inefficiency of available overexpression systems in inducing *PVT1* circularization [17, 70, 71]. Overcoming these technical challenges in future studies will be crucial for gaining a clearer understanding of the significance of these interactions.

In conclusion, our study revealed that *circPVT1* plays a pro-fibrotic function in cardiac fibrosis, regulating TGF-β signaling and cardiac fibroblast activation. *circPVT1* binds fibrosis-related miRNAs miR-125b-5p and miR-30a-5p, potentially influencing their cellular bioavailability.

Although further studies are needed to fully understand the spatial and dynamic aspects of *circPVT1*-mediated regulation, including its potential to modulate miRNA activities through localized interactions, our findings highlight the importance of *circPVT1* in cardiac fibrosis. These insights may open new avenues for research aimed at exploiting *circPVT1* as a novel therapeutic target to reduce fibrosis and counteract adverse cardiac remodeling.

## Methods and materials

### Cell culture

AC16 human cardiomyocyte cell line (Merck KGaA, Darmstadt, Germany) was cultured in DMEM/F12 containing 2 mM L-Glutamine, 12.5% FBS and 1X Penicillin-Streptomycin Solution. HUVEC (Gibco, Thermo Fisher Scientific Inc, Waltham, MA, USA) were cultured in EGM™-2 Endothelial Cell Growth Medium-2 BulletKit (Lonza, Basel, Switzerland). HCF (PromoCell, Heidelberg, Germany) were cultured in PromoCell Fibroblast Growth Medium 3 (PromoCell, Heidelberg, Germany) containing the Supplement Mix Growth Medium Kit: Fetal Calf Serum: 0.1 mL/mL, Basic Fibroblast Growth Factor (recombinant human) 1 ng/mL and Insulin 5 µg/mL (PromoCell, Heidelberg, Germany). Cells used for experiments were passaged with a split ratio of 1:3. All the cell lines were tested for mycoplasma contamination.

### Cell transfection

siRNAs targeting the BSJ of *circPVT1* (siRNA *cPVT1*) or linear *PVT1* (siRNA *linPVT1*) were purchased from Eurofins Genomics (Ebersberg, Germany) (Supplementary Table S3) and transfected into AC16 and HCF cells at a final concentration of 50 nM. The ON-TARGET plus non-targeting pool siRNA (Dharmacon, Lafayette, LA, USA) was used as a negative control. The inhibitors of miR-125b-5p and miR-30a-5p and the corresponding negative control were purchased from Qiagen (Hilden, Germany) and transfected into HCF cells at a final concentration of 50 nM. siRNA transfection reagent supplied by Santa Cruz Biotechnology (Dallas, TX, USA) was used for cell transfections according to the manufacturer’s protocol. HCF cells were treated with 10 ng/mL TGF-β1 48 h after siRNA/miRNA transfection (Merck KGaA, Darmstadt, Germany).

### Patient selection and tissue collection

LV end-stage IHF biopsies were harvested at the Department of Heart Failure and Transplantology, Cardinal Stefan Wyszyński Institute of Cardiology, Warszawa, Poland, from end-stage IHF patients undergoing heart transplantation. The healthy human LV was obtained from age- and sex-matched organ donor patients whose hearts were not used for transplantation due to technical reasons (e.g., donor/recipient incompatibility) and collected at the Department of Heart Failure and Transplantology, Department of Mechanical Circulatory Support and Transplant, National Institute of Cardiology, Warszawa, Poland. The donors had no relevant cardiology history or abnormalities in ECG or echocardiography. Samples were rinsed immediately in saline, blotted dry, frozen in liquid nitrogen, and kept at −80 °C until further processing. The protocol was authorized by Terenowej Komisji Bio-etycznej Przy Instytucie Kardiologii-Warsaw Ethics Committee, protocol number: IK-NPIA-0021-14/1426/18. IHF patients and control characteristics are described in Supplementary Table S4. There is no blinding of researchers or participants.

### AMI mouse model

All experimental procedures adhered to the Italian National Institutes of Health Guidelines and the Guide for the Care and Use of Laboratory Animals (Institute of Laboratory Animal Resources, National Academy of Sciences, Bethesda, MD). These procedures were approved by the Institutional Animal Care and Use Committee (IACUC 1115, authorization 903/2020-PR). The sample size for animal experiments was determined by power analysis and adjusted based on prior studies and pilot data. Subsequently, animals were randomized into different groups. No blinding was performed. Prior to all surgical and perfusion procedures, mice were anesthetized with an intraperitoneal injection of 10 mg/kg xylazine (Intervet Farmaceutici, Milan, Italy) and 100 mg/kg ketamine (Ketavet 100; Intervet Farmaceutici, Milan, Italy). One day before and 7 days after the surgical procedure, acetaminophen 1 mg/mL was administrated in drinking water as an analgesic drug. Myocardial infarction was induced in 8–12-week-old C57BL/6N female mice by coronary artery ligation under anesthesia, with the mice mechanically ventilated. Briefly, the thoracotomy was performed via the third left-intercostal space and the left coronary artery was ligated. The chest was closed, and the mice were allowed to recover. Sham-operated mice underwent a similar surgical procedure, except for the ligature around the coronary artery, which was not tied. Transthoracic echocardiography was performed using a high-performance ultrasonographic Imaging System (Vevo 2100; FUJIFILM Visualsonics Inc., Toronto, ON, Canada).

### Western blotting

Total proteins were extracted from HCF cells using cold RIPA buffer (Thermo Fisher Scientific Inc., MA, USA) with freshly added 1X Protease and Phosphatase Inhibitor Cocktail (Thermo Fisher Scientific Inc., MA, USA), followed by centrifugation at 12,000 rpm at 4 °C for 20 min. After quantification with the BCA protein assay kit (Thermo Fisher Scientific Inc., MA, USA), protein samples (40 µg) were separated on 4-12% Bis-Tris precast polyacrylamide gels (Thermo Fisher Scientific Inc., MA, USA) and subsequently transferred to PVDF membranes. Transferred membranes were first blocked with 5% dried milk in TBS buffer (20 mM Tris, 500 mM NaCl, pH 7.5) containing 0.1% of Tween-20 for 1 h at room temperature to block non-specific binding sites. This was followed by an overnight incubation at 4°C with primary antibodies to α-SMA (1A4, 1:200, Santa-Cruz Biotechnology, Dallas, TX, USA), CTGF (E-5, 1:200, Santa-Cruz Biotechnology, Dallas, TX, USA), phospho-SMAD2 (Ser465/467)/SMAD3 (Ser423/425) (8828, 1:500, Cell Signaling Technology, Danvers, Massachusetts, USA), SMAD2/3 (8685, 1:1000 Cell Signaling Technology, Danvers, Massachusetts, USA) and β-actin (ab8227, 1:2500, Abcam, Cambridge, UK) diluted in TBS buffer containing 2.5% BSA and 0.1% Tween-20. The next day, membranes were incubated for 1 h at room temperature with HRP-conjugated secondary antibodies (anti-mouse IgG HRP-conjugated NA931 and anti-rabbit IgG HRP-conjugated NA934, Cytiva, 1:1000 dilution) in TBS buffer containing 2.5% BSA and 0.1% Tween-20. Protein bands were detected using SuperSignal™ West Pico PLUS Chemiluminescent Substrate (Thermo Fisher Scientific Inc., MA, USA) and visualized using Odyssey Fc apparatus (LI-COR Biosciences, Nebraska, USA). The intensity signal of each band was compared to the β-actin signal.

### RNA isolation and RT-qPCR

Following the manufacturer’s instruction, total RNA was extracted from cells using TRIzol reagent (Thermo Fisher Scientific Inc., MA, USA). Quantity, quality and integrity of the extracted RNAs were checked by Nanodrop ND-1000 (Thermo Fisher Scientific Inc., MA, USA) and Bioanalyzer 2100 (Agilent Technologies, CA, USA), respectively.

RNA was reverse transcribed to cDNA with random hexamers using the GoScript Reverse Transcription System (Promega Corporation, WI, USA). RT-qPCR was performed using a SYBR green PCR kit (GoTaq qPCR Master Mix, Promega Corporation, WI, USA) according to the manufacturer’s instructions on a StepOne Plus Instrument (Thermo Fisher Scientific Inc., MA, USA). Relative RNA expression was calculated using the 2-ΔΔCt method [72], normalizing to the average expression levels of UBC and RPL23. The primers sequences used are listed in Supplementary Table S5.

miRNAs were reverse transcribed using Applied Biosystems™ TaqMan™ MicroRNA Reverse Transcription Kit and their expression levels were measured using TaqMan MicroRNA single assays and TaqMan™ Universal PCR Master Mix following the manufacturer’s instructions. Thermo Fisher Scientific Inc., MA, USA, provided all reagents. Real-time PCR was performed on Step-One plus real-time PCR System (Thermo Fisher Scientific Inc., MA, USA). Relative RNA expression was calculated using the 2-ΔΔCt method [72] and U6 and miR-16 were used as internal controls for miRNAs.

### RNase R assay

1 µg of RNA was incubated with 10 U of RNase R (LGC Biosearch Technologies, Steinach, Germany) and 10 U of RiboLock RNase Inhibitor (Thermo Fisher Scientific Inc., MA, USA) in 1× RNase R buffer in a 10 µL reaction at 37 °C for 30 min, followed by heat inactivation at 95 °C for 3 min. For comparison, samples were also incubated under the same conditions without RNase R.

### Subcellular fractionation

Nuclear and cytoplasmic fractions from AC16 cells were separated by using a PARIS™ Kit (Thermo Fisher Scientific Inc., MA, USA). 1 ×10^7^ cells were re-suspended in 300 µL ice-cold Cell fractionation buffer, incubated on ice for 10 min, and then centrifuged for 5 min at 4 °C at 500 × *g* to separate the nuclear and the cytoplasmic cell fractions. The supernatant containing the cytoplasmic fraction was collected. The nuclear fraction pellet was washed in ice-cold Cell fractionation buffer and then lysed in 300 µL Cell Disruption Buffer.

### Absolute quantification

ddPCR was performed using the QX200 Droplet Digital PCR System. Briefly, 25 ng of cDNA obtained with GoScript™ Reverse Transcriptase (Promega A5003), 1x QX200™ ddPCR™ EvaGreen Supermix (Bio-Rad 1864034), and 125 nM of both forward and reverse primers were mixed in a final volume of 20 μL. Twenty microliters of each reaction mix were converted into droplets using the QX200 droplet generator 1260T (Bio-Rad). Droplet-partitioned samples were then transferred to a 96-well plate, sealed, and cycled in a T100 Thermal Cycler (Bio-Rad) under the following cycling protocol: 95 °C for 5 min, followed by 45 cycles of 95 °C for 30 s and 58 °C for 1 min. For signal stabilization, samples were kept at 4 °C for 5 min, then at 90 °C for 5 min, followed by an indefinite hold at 4 °C. A PCR ramp rate of 2 °C/sec was selected for the protocol. The cycled plate was then transferred and read using the QX200 reader (Bio-Rad).

### Circular RNA pull-down assay

Biotin-labeled control oligonucleotide (NEG CTRL-ASO) (5’-GCTGGTAGAGGGAGCAGATG-3’[BtnTg]) [73] and an oligonucleotide complementary to the *circPVT1* BSJ sequence (*circPVT1*-ASO) (5’-AAAAGATCAGGCCTCAAGC-3’[BtnTg]) were synthesized by Eurofins Genomics (Ebersberg, Germany) and used for circRNA pull-down assay, as described by Das et al. [73]. Briefly, 2.5 ×10^6^ HCF cells were lysed with 1 mL of ice-cold polysome extraction buffer (20 mM Tris-HCl, pH 7.5, 100 mM KCl, 5 mM MgCl2 and 0.5% Nonidet P-40) supplemented with 40 U RNase inhibitor (Promega Corporation, WI, USA) and 5 μL of 20x protease inhibitor (Thermo Fisher Scientific Inc., MA, USA), and centrifuged at 12,000 × *g* for 10 min at 4 °C. The supernatant was incubated on a tube rotator with 1 μL of 100 μM *circPVT1*-ASO or NEG CTRL-ASO overnight at 4 °C. The next day, 50 μL of Dynabeads Streptavidin M-280 (Thermo Fisher Scientific Inc., MA, USA), 40 U of RNase inhibitor (Promega Corporation, WI, USA), and 5 μL of 20x protease inhibitor (Thermo Fisher Scientific Inc., MA, USA) were added to the mixture and incubated for 90 min on a tube rotator. After 5 washing steps, TRIzol reagent (Thermo Fisher Scientific Inc., MA, USA) was added for RNA extraction.

### microRNA pull-down assay

Biotinylated miR-125b-5p (5’-UCCCUGAGACCCUAACUUGUGA-3’[BtnTg]), miR-30a-5p (5’- UGUAAACAUCCUCGACUGGAAG-3’[BtnTg]) and negative control (5’-UCACAACCUCCUAGAAAGAGUAGA-3’[BtnTg]) oligonucleotides were synthesized by Eurofins Genomics (Ebersberg, Germany) and used for miRNA pull-down assays. Briefly, 1 ×10^6^ HCF cells were transfected with 50 nM biotinylated miRNAs and harvested 48 h after transfection. Cells were lysed with 700 μL of ice-cold lysis buffer (20 mM Tris-HCl, pH 7.5, 100 mM KCl, 5 mM MgCl2 and 0.5% Nonidet P-40) supplemented with 40 U of RNase inhibitor (Promega Corporation, WI, USA) and 5 μL of 20x protease inhibitor (Thermo Fisher Scientific Inc., MA, USA), incubated on ice for 20 minutes and then centrifuged at 10,000 × *g* for 15 min at 4 °C. Next, the cell lysates were incubated with 50 μL of Dynabeads Streptavidin M-280 (Thermo Fisher Scientific Inc., MA, USA), 40 U of RNase inhibitor (Promega Corporation, WI, USA) and 5 μL of 20x protease inhibitor (Thermo Fisher Scientific Inc., MA, USA) on a tube rotator overnight at 4 °C. To minimize non-specific binding of RNA, beads were incubated with yeast tRNA (Thermo Fisher Scientific Inc., MA, USA) for 3 h at 4 °C before being incubated with cell lysates. After 5 washing steps, bound RNA was extracted from magnetic beads using TRIzol reagent (Thermo Fisher Scientific Inc., MA, USA).

### Secondary structure analysis

Publicly available SHAPE-MaP reactivity data for circ/linear *PVT1* were retrieved from GEO (GSM3593181, GSM3593215) [30]. Raw reactivities were normalized by performing a box-plot normalization scheme (i.e., dividing by the mean of the top 10% of reactivities, after excluding instances above 1.5 times the interquartile range) [74], separately for each molecule. SHAPE-MaP-informed, MFE-based (Minimum Free Energy) predictions were performed with RNAfold [75] by using the default configuration, setting “assume RNA molecule to be circular” in the case of *circPVT1*, and providing normalized reactivity scores for the circular and linear RNAs. Forna [76] was utilized for visualization of the predicted structures and the per-base probability of the observed bound/unbound states (i.e., the probability that each nucleotide is paired or unpaired in the structure under study). R4RNA R package [77] was employed for creating corresponding arc-plots highlighting the MREs of interest.

### RNA sequencing and bioinformatics analysis

For long RNA sequencing analysis, total RNA was isolated with TRIzol reagent (Thermo Fisher Scientific Inc., MA, USA), followed by RNA purification using the Zymo RNA Clean & Concentrator™ kit (Euroclone, Milan, Italy) according to the manufacturer’s instructions. RNA samples were quantified, and quality tested by Agilent 2100 Bioanalyzer RNA assay (Agilent Technologies, Santa Clara, CA) and Caliper LabChip GX (PerkinElmer, Waltham, MA). Universal Plus mRNA-Seq kit (Tecan Genomics, Redwood City, CA) was used for library preparation following the manufacturer’s instructions (library type: stranded strinfr-secondstrand). Final libraries were checked with Qubit 2.0 Fluorometer (Invitrogen, Carlsbad, CA) and Agilent Bioanalyzer DNA assay or by Caliper LabChip GX (PerkinElmer, Waltham, MA). Libraries were then prepared for sequencing and sequenced on paired-end 150 bp mode on NovaSeq 6000 (Illumina, San Diego, CA) to a depth of 50 M reads. After trimming sequencing adapters and low-quality sequences with ERNE software [78], the quality was checked using RSeqQC6 package [79]. STAR aligner [80] was used to align reads to the hg38 human genome. Transcript abundances were produced usingStringtie [81]. DESeq2 [82, 83] was then used to compare gene expression levels and transcripts by fitting a Generalized Linear Model (GLM) for each gene. All libraries were prepared and sequenced at the Institute for Applied Genomics (IGA) in Udine (Italy). The Gene Ontology analysis for biological processes and molecular functions was performed using Enrichr [84] (March 29, 2021, https://maayanlab.cloud/Enrichr/) with default parameters.

Small RNAs derived from *circPVT1* pull-down samples were processed using the *SMARTer small RNA* kit from Clontech (Illumina, CA, USA) following the manufacturer’s instructions and sequenced on a *Novaseq 6000* Illumina platform to obtain 10 M reads per sample on average of 100 bp single end reads. After demultiplexing, reads were trimmed using cutadapt to remove polyA tracts (-a AAAAAAAA), low-quality bases (-q 15), and a prefix of three bases (-u 3). Trimmed reads were then aligned using bowtie against the miRBase v22 fasta collection of mature miRNAs with the following parameters (-k 1 -n 1 -l 8 -a --best --strata). Counts onto genome annotation (miRBase) were obtained using samtools idxstats. Then, the matrix of raw counts was introduced into R and analyzed with limma. Briefly, miRNAs were expressed if a minimum of 5 reads was present in at least *n* = 4 samples, producing a matrix of *n* = 792 miRNAs. Then, normalization was obtained using the TMM method implemented in the calcNormFactors function in the edgeR R package and transformed using the voom function from limma [85]. Differentially expressed miRNAs were detected with an FDR < 0.01 (BH correction) and average expression across all samples >5. miRNA sequencing was performed at the Center for Omics Sciences, IRCCS Ospedale San Raffaele, Milan, Italy.

### miRNA target prediction

mirDIP 5.2 [31] (https://ophid.utoronto.ca/mirDIP) integrating predictions across multiple resources and Tarbase v9.0 [33] (https://dianalab.e-ce.uth.gr/tarbasev9), a database of experimentally supported miRNA targets, were used to identify miRNA targets.

### Statistical analysis

GraphPad Prism 7.01 software (GraphPad Software Inc., San Diego, CA, USA) was used for statistical analysis and for graph generation. A normality test was initially carried out, and a two-tailed S tudent’s *t* test, or Mann–Whitney, was used to identify statistically significant differences as appropriate. ANOVA or t-test corrected for multiple comparisons was used as appropriate. *P*-value of <0.05 was considered statistically significant. Values were expressed as mean ± standard error of the mean (SEM). Sample size was determined based on prior analyses and studies, maintaining a minimum sample size of at least *n* = 3 for all experiments.

### Original data

Original data of western blots are reported as Original western blots.

## Supplementary information

Supplementary Figures

Supplementary Table S1

Supplementary Table S2

Supplementary Table S3

Supplementary Table S4

Supplementary Table S5

Original western blots

## Data Availability

RNA sequencing raw and processed data were deposited at GEO under accession number GSE262531.
