# Supplementary material for: Circular PVT1 promotes cardiac fibroblast activation interacting with miR-30a-5p and miR-125b-5p
Source: Cell Death Dis. 2025 Apr 21;16(1):325. doi: 10.1038/s41419-025-07652-7 (PMC12012019; doi:10.1038/s41419-025-07652-7)
Supplement: Supplementary file 1 — Supplementary Figures [file 41419_2025_7652_MOESM1_ESM.pdf]

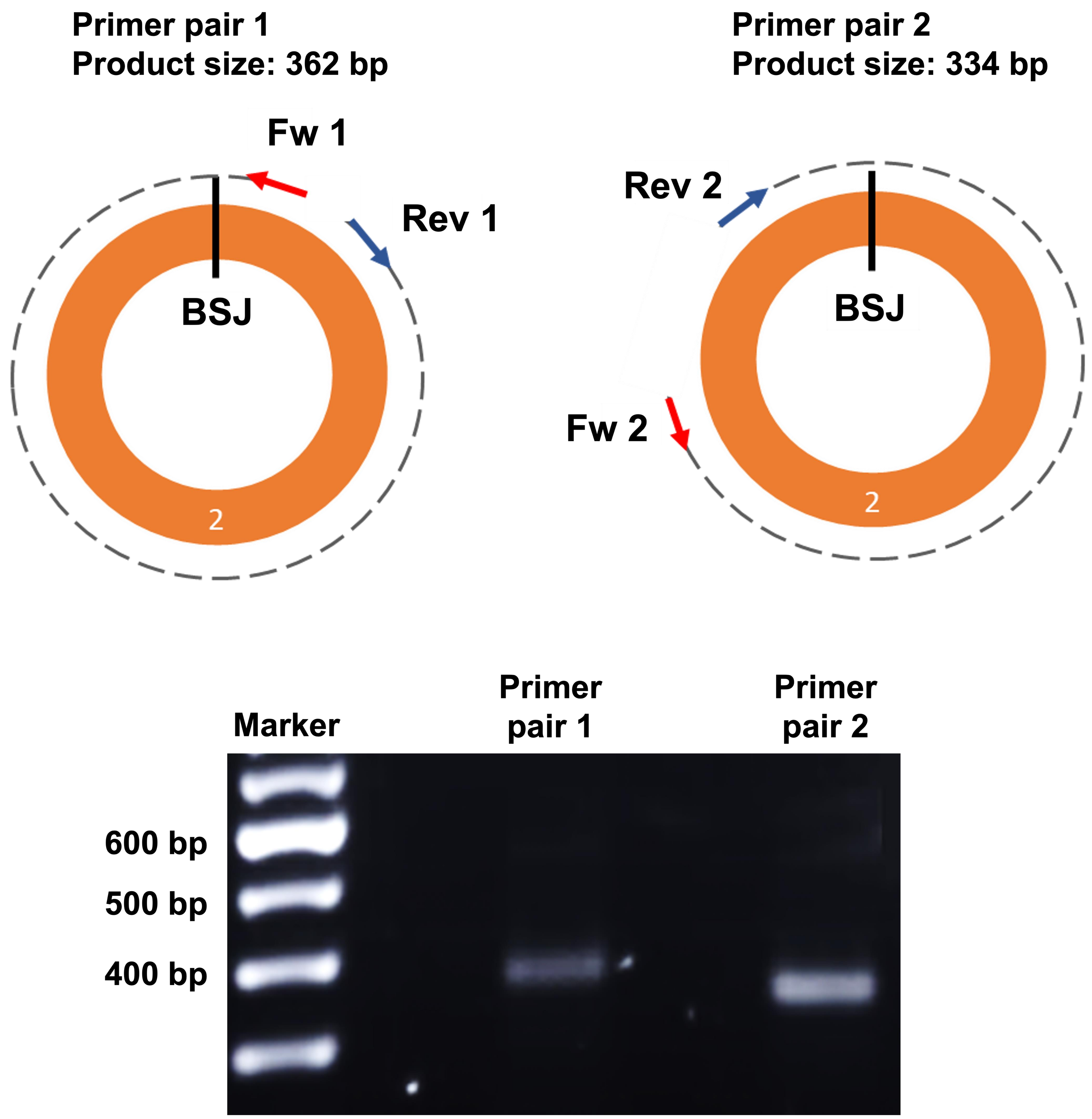

Figure S1. Characterization of *circPVT1* sequence and BSJ. Two divergent primer pairs were used to amplify the *circPVT1* sequence in an LV human sample. PCR products were visualized on agarose gel. Agarose gel shows two bands representing cDNA fragments amplified with primer pair 1 (362 bp) and primer pair 2 (334 bp).

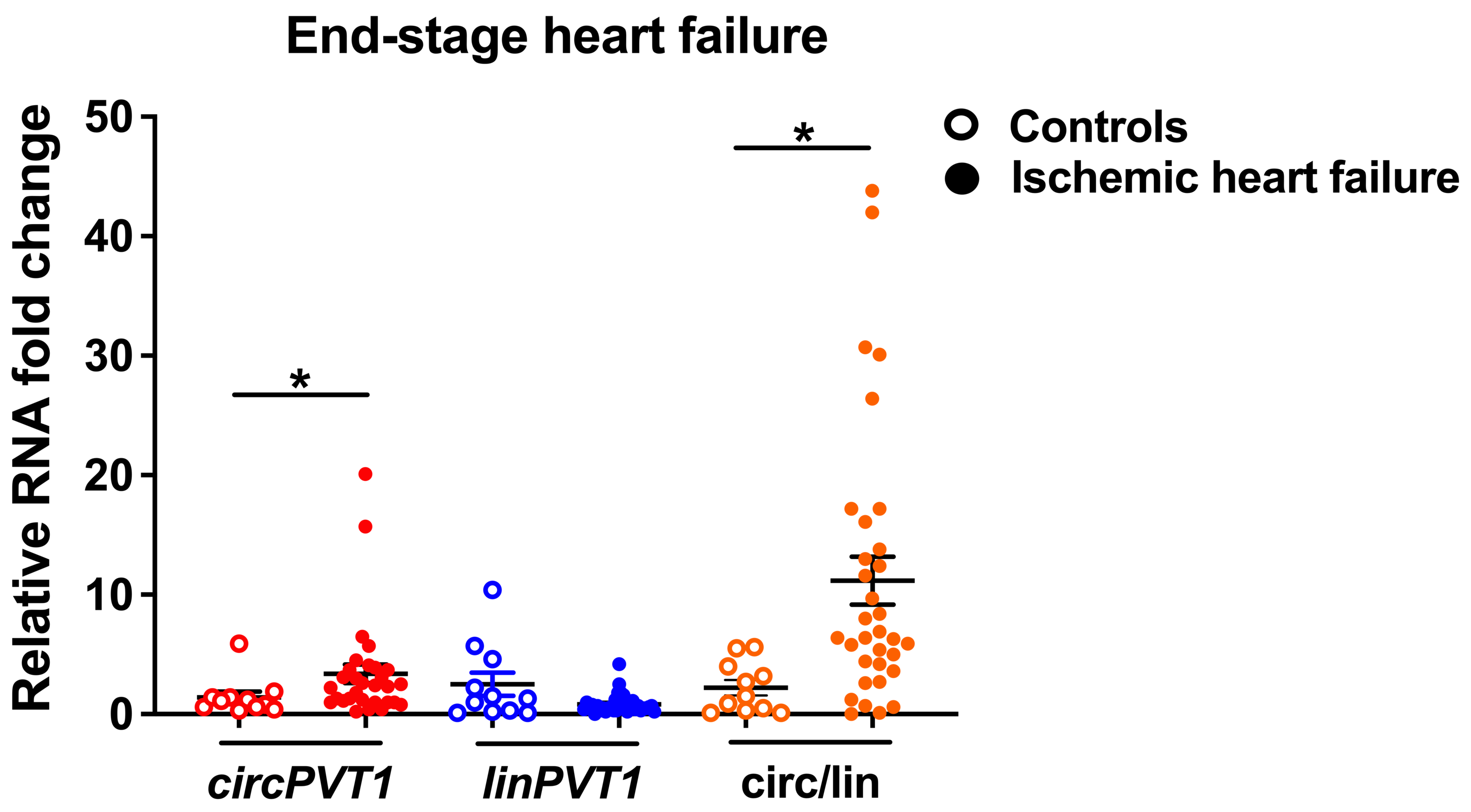

Figure S2. *circPVT1* is increased in end-stage IHF patients. Total RNA was extracted from LV tissue derived from end-stage IHF patients (n = 31) and age and sex matched control subjects (n = 11). Dot-plots show *circPVT1* and host gene relative expression measured by RT-qPCR and expressed as fold change. Circular-to-linear ratio (circ/lin) indicates the circRNA enrichment compared to the host gene (*linPVT1*). Data are indicated as mean ± SEM (*$p < 0.05$).

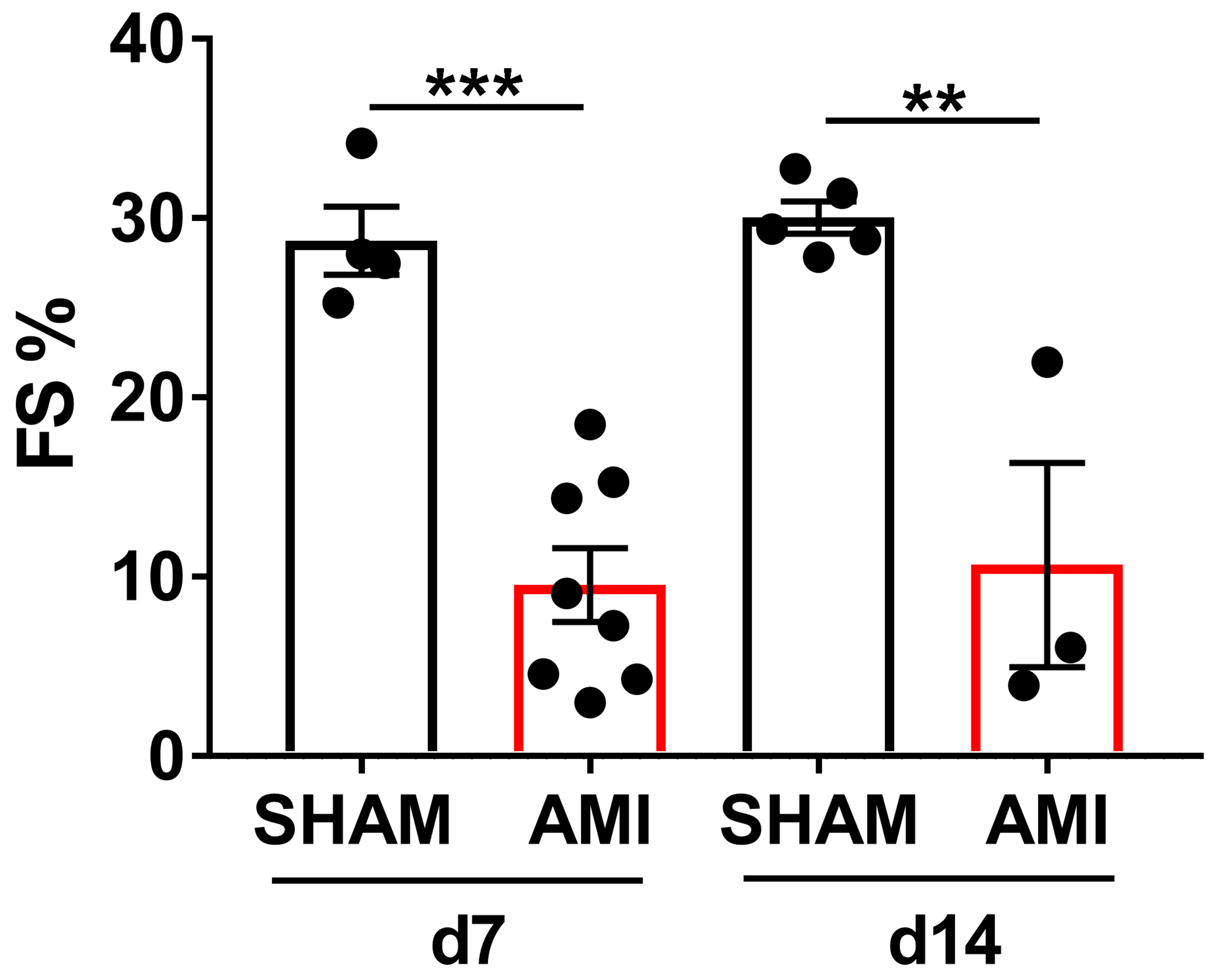

Figure S3. Fractional shortening is decreased in an AMI mouse model. The percentage of fractional shortening (FS %) in mice at 7 (*n* = 8) and 14 (*n* = 3) days post-AMI. Sham-operated mice served as controls (SHAM 7 days, *n* = 4; SHAM 14 days, *n* = 5). Data are presented as mean ± SEM (**$p < 0.01$, ***$p < 0.001$).

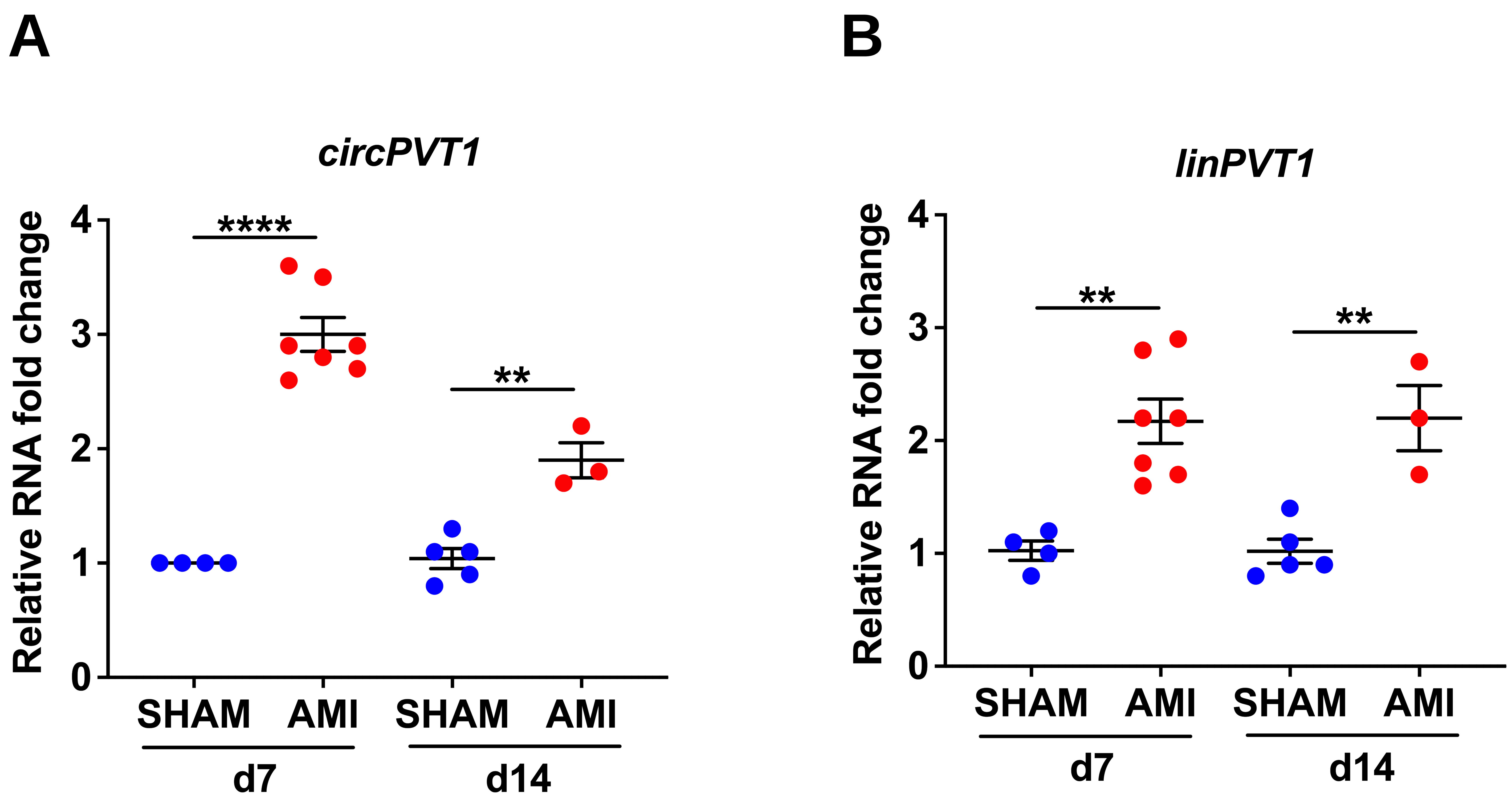

Figure S4. *circPVT1* and linear *PVT1* levels are increased in AMI mice. RT-qPCR analysis of *circPVT1* **(A)** and linear *PVT1* **(B)** expression 7 (n = 8) and 14 (n = 3) days after AMI in mice. Sham-operated mice served as controls (SHAM 7 days, n = 4; SHAM 14 days, n = 5). Fold change values are presented as mean ± SEM (**$p < 0.01$, ****$p < 0.0001$).

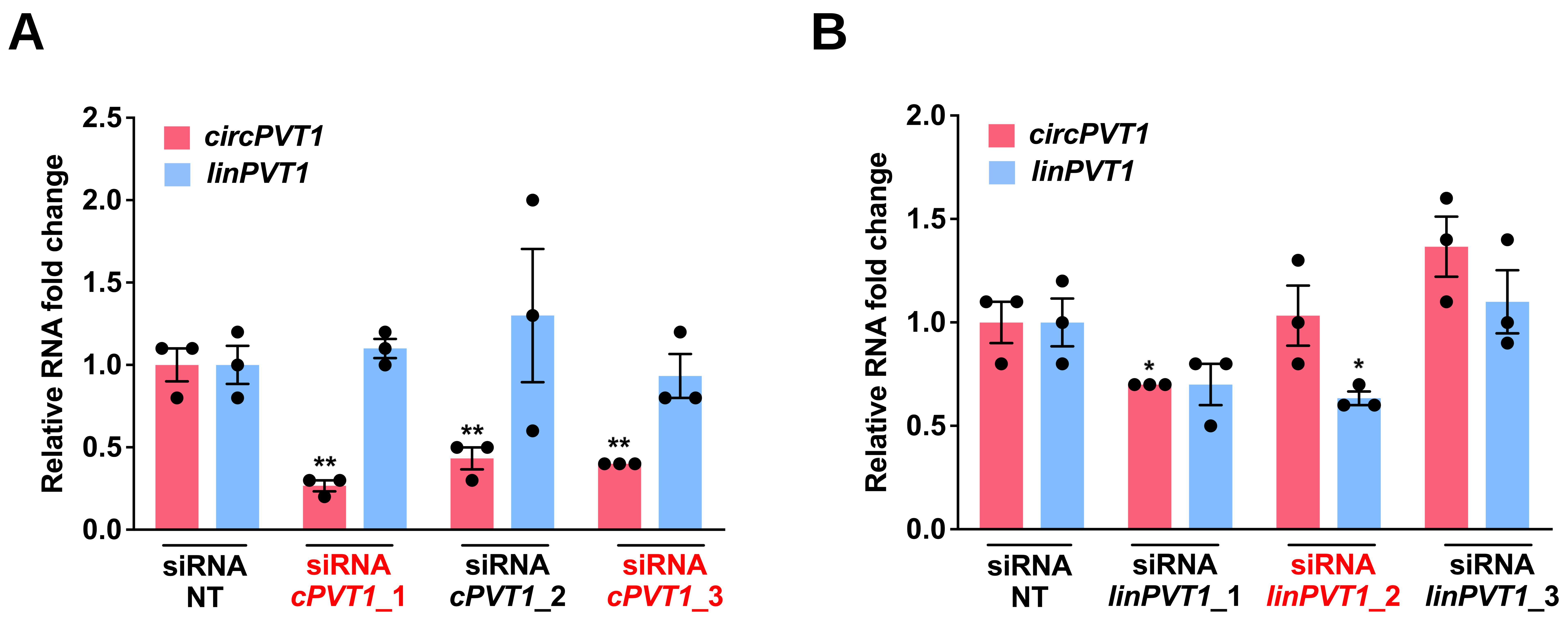

Figure S5. siRNA-screening for transcript-specific and efficient knockdown of *circPVT1* and linear *PVT1*. siRNAs targeting the BSJ were designed for the silencing of *circPVT1* **(A)** and siRNAs targeting exons different from exon 2 were designed to silence linear *PVT1* **(B)**. A non-targeting siRNA (siRNA NT) was used as a negative control. siRNAs were transfected in AC16 cardiomyocyte cells and the levels of the circular and linear forms of *PVT1* were analyzed by RT-qPCR 72 hours later. Scatterplots show circular and linear *PVT1* expression levels as fold change. siRNA *cPVT1*_1, siRNA *cPVT1*_3, and siRNA *linPVT1*_2 were chosen for *circPVT1* and linear *PVT1* knockdown, respectively (in red), as they elicited the most significant changes. Lines indicate mean and SEM values for each group (n = 3 independent biological replicates, $*p < 0.05$, $**p < 0.01$).

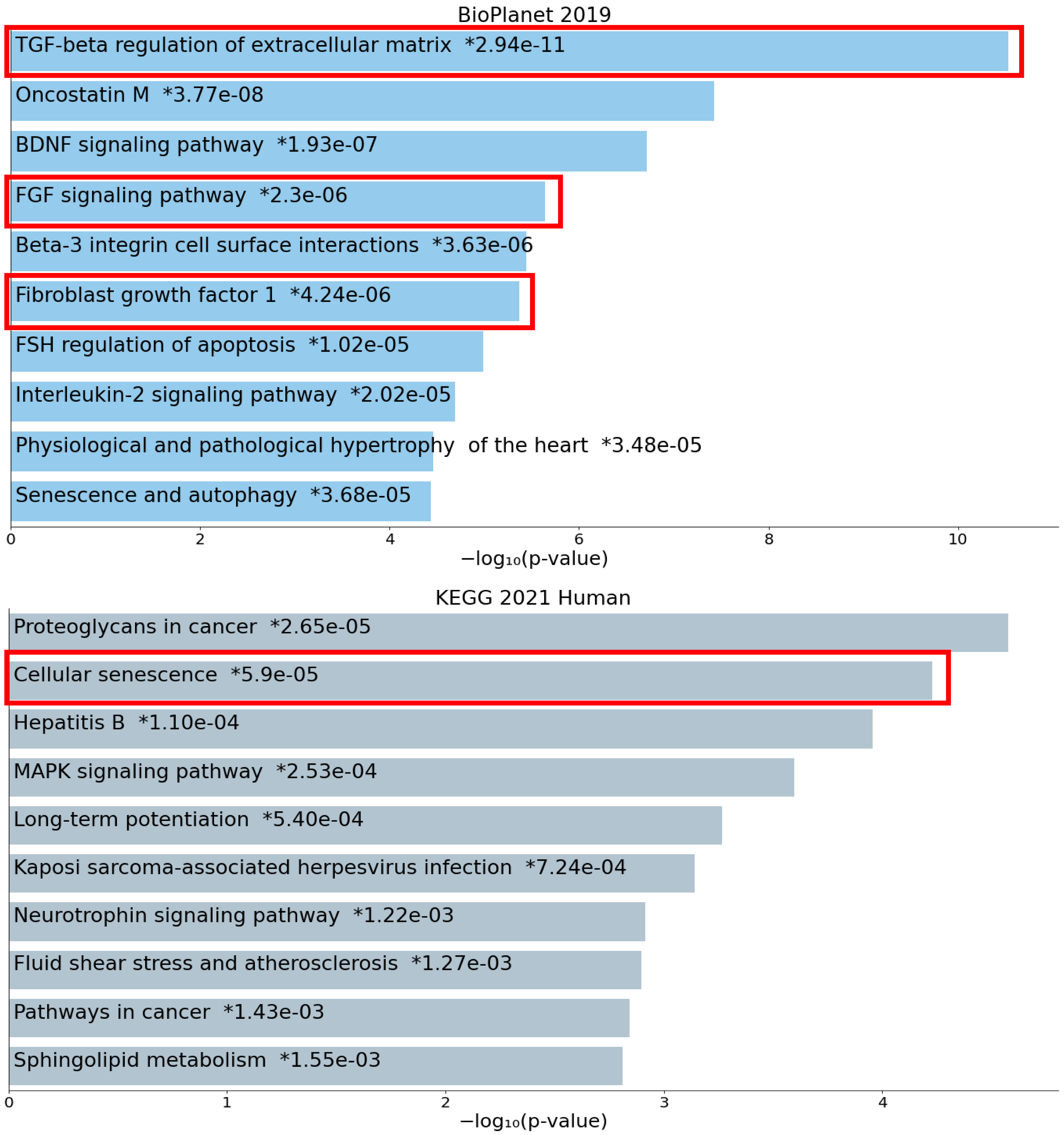

Figure S6. The knockdown of *circPVT1* affects genes involved in extracellular matrix formation, fibrosis, and cellular senescence. Top enriched pathways of significantly differentially expressed genes (FDR < 0.01) upon *circPVT1* knockdown using siRNA *cPVT1*_1. Red boxes highlight prominently regulated cellular processes potentially involved in HF pathomechanisms. Bars are sorted by p-value ranking.

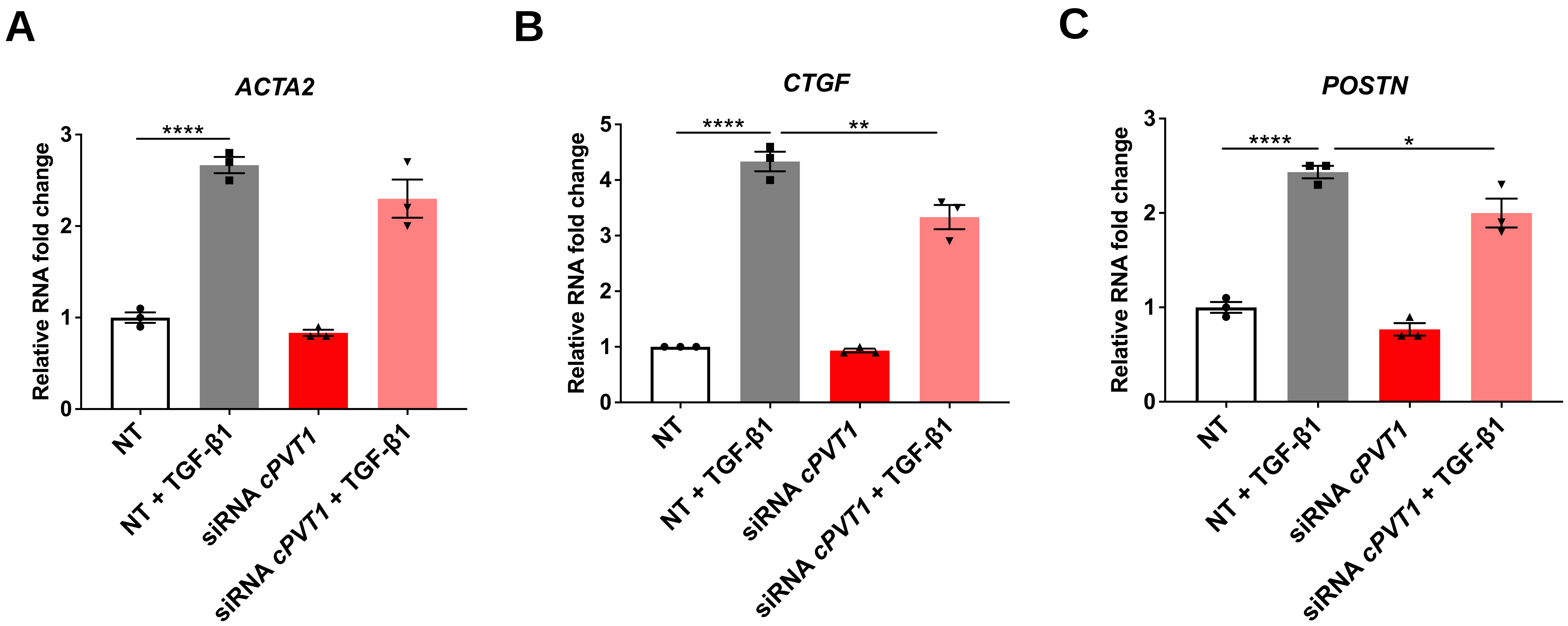

Figure S7. The knockdown of *circPVT1* reduces TGF-β1-induced expression of pro-fibrotic markers. HCF were transfected with non-targeting siRNA (NT) or *circPVT1*_3 siRNA (siRNA *cPVT1*) and then stimulated with TGF-β1 for 24h. RT-qPCR analysis of *ACTA2* **(A)**, *CTGF* **(B)**, and *POSTN* **(C)** expression mean values are shown in fold change ± SEM (n = 3 independent biological replicates, *$p < 0.05$, **$p < 0.01$, ****$p < 0.0001$).

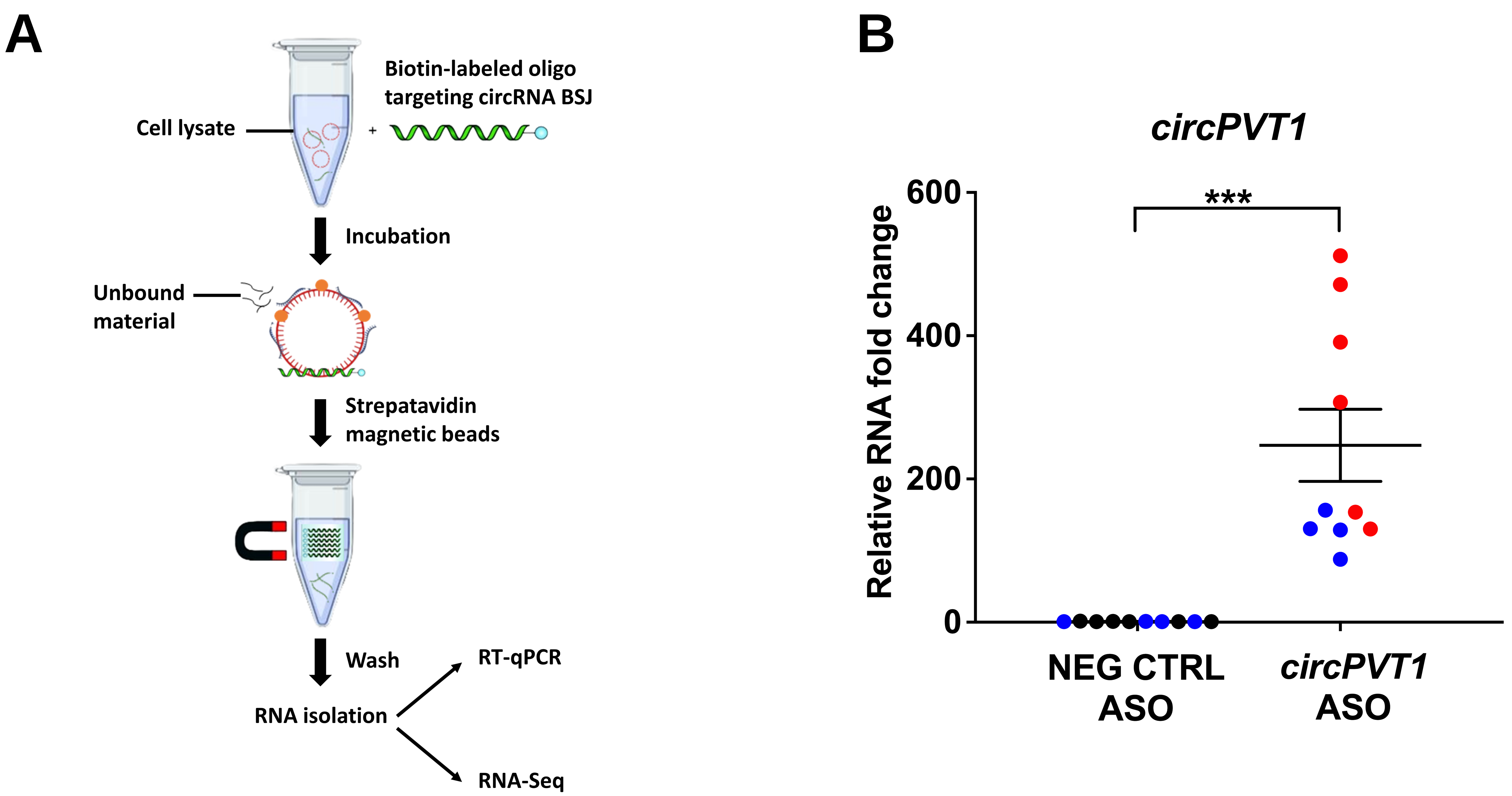

Figure S8. ***circPVT1*** **enrichment in pull-down experiments. (A)** Schematic illustration of circRNA pull-down assay. Biotinylated ASOs targeting the *circPVT1* back-splicing junction (*circPVT1* ASO) and biotinylated non-targeting control oligonucleotides (NEG CTR ASO) were used in pull-down experiments against HCF lysates. RT-qPCR evaluated relative expression levels of *circPVT1* **(B)**. Blue dots indicate samples used for RNA-sequencing profiling. Mean values and error bars are presented in fold change ± SEM (n = 10 independent biological replicates, ***p < 0.001).

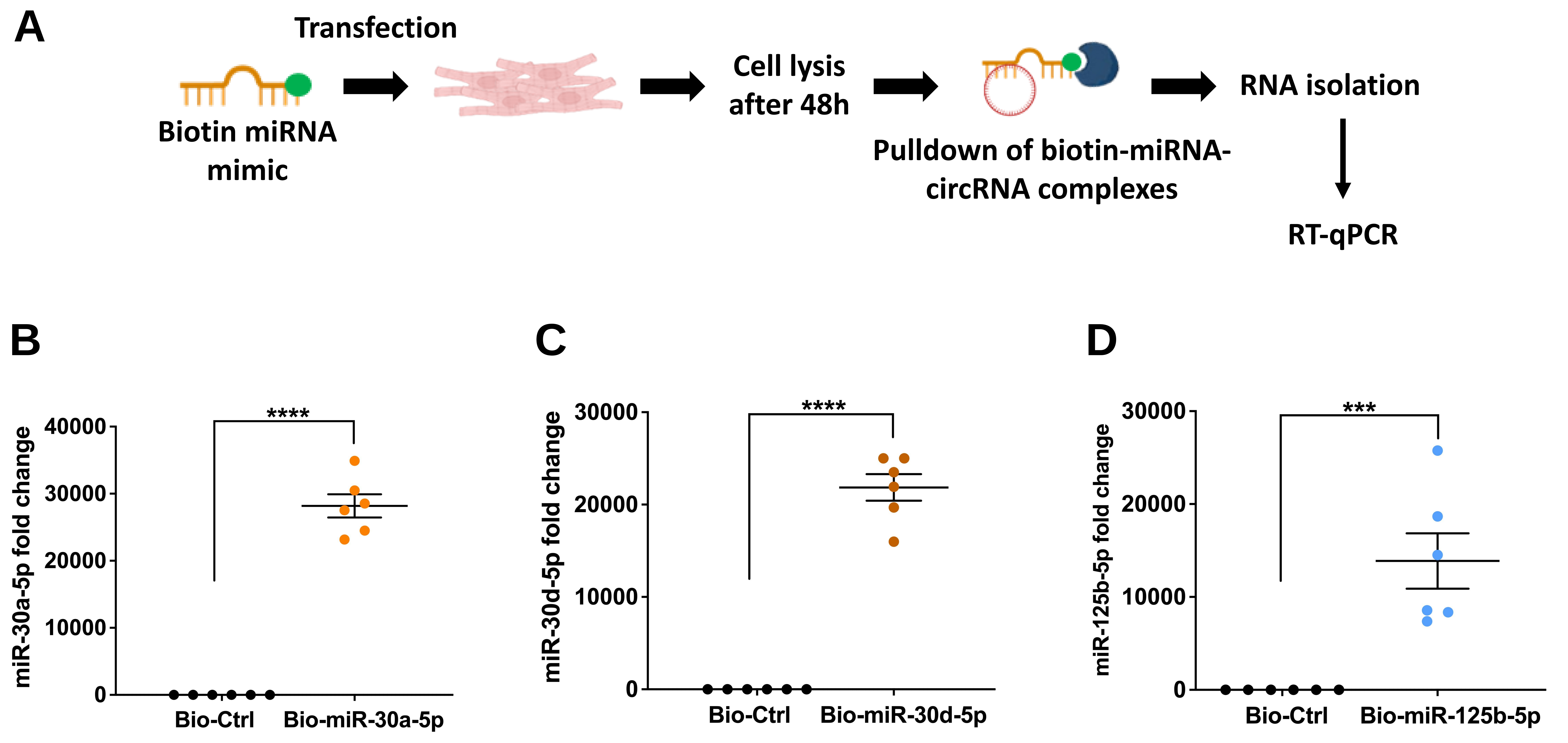

Figure S9. miR-30a-5p, miR-30d-5p, and miR125b-5p enrichment in miRNA pull-down. **(A)** Schematic illustration of miRNA pull-down assay. Biotinylated miRNAs (bio-miR-30a-5p, bio-miR-30d-5p and bio-miR-125b-5p) or biotinylated miRNA control (bio-Ctrl) were transfected in HCF and then pulled-down using streptavidin-coupled beads. Relative expression levels of miR-30a-5p **(B)**, miR-30d-5p **(C)** and miR-125b-5p **(D)** were evaluated by RT-qPCR. Mean values are indicated in fold change ± SEM (n = 6 independent biological replicates, ***$p < 0.001$, ****$p < 0.0001$).

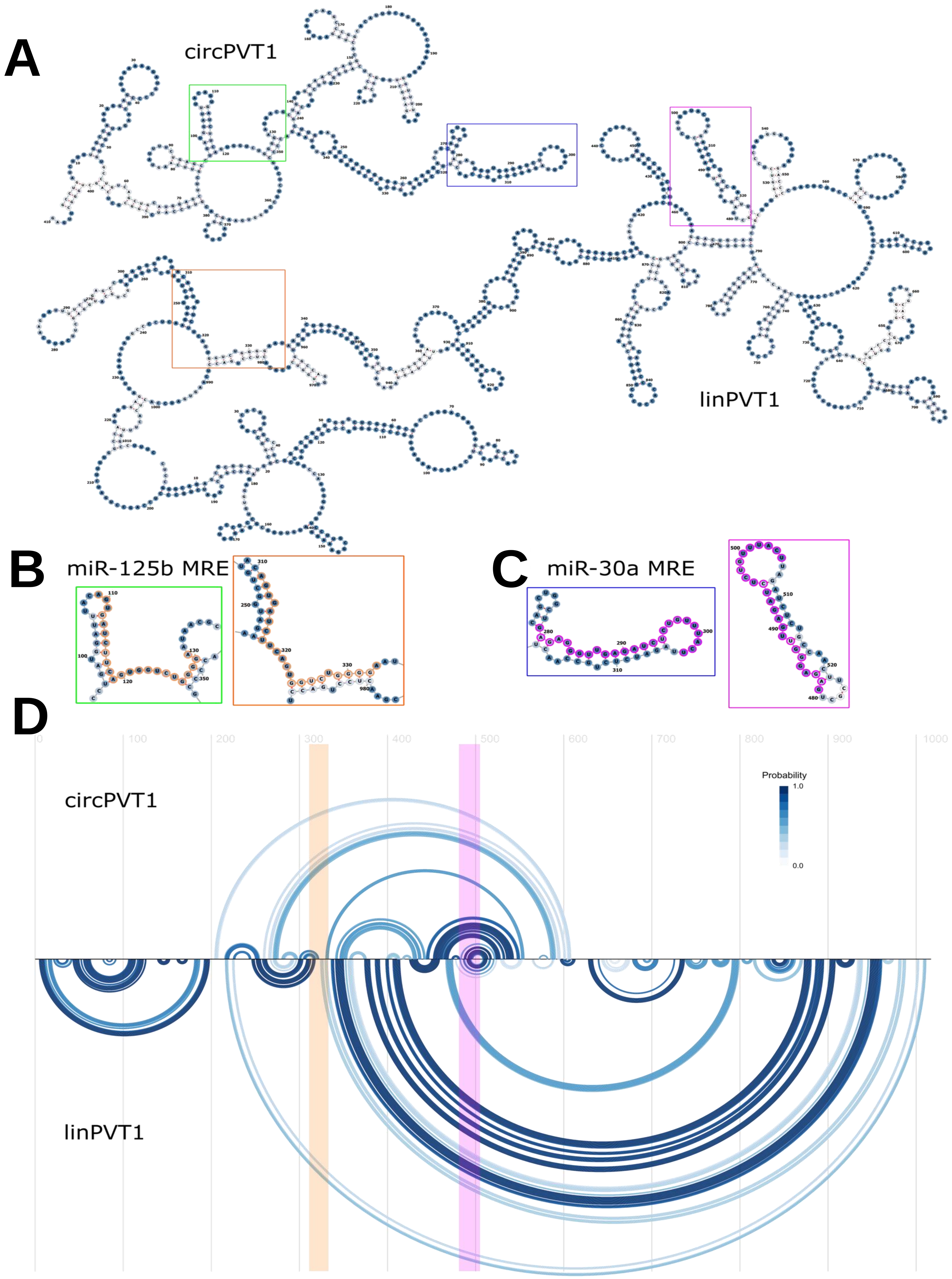

Figure S10. Comparison of the secondary structures of circular and linear *PVT1*. (A) SHAPE-MaP-informed RNAfold prediction of the conformation of the entire sequence of *circPVT1* (top) and linear *PVT1* (bottom). Colored boxes indicate MRE-containing loci that are explored in finer detail in the following panels (green: *circPVT1*-miR-125b-5p MRE; blue: *circPVT1*-miR-30a-5p; orange: *linPVT1*-miR-125b-5p MRE; purple: *linPVT1*-miR-30a-5p). **(B)** Zoomed-in aspect of the locus harboring predicted MREs against miR-125b-5p on the circular (green; 109-131 bp) and linear (311-333 bp) forms. MREs are highlighted in orange. **(C)** Zoomed-in aspect of the locus harboring predicted MREs against miR-30a-5p on the circular (279-303 bp) and linear (481-505 bp) forms. MREs are highlighted in purple. Bases are colored according to the per-base probability of the observed state (i.e., paired or unpaired), as derived from the RNAfold analysis (color range: 0 (white) to 1 (steel blue)). **(D)** Arc plots juxtaposing the circular (top) and linear (bottom) structural predictions, highlighting binding probabilities in the same white-to-blue combination as in (**B-C**). Predicted MRE sites for miR-125b-5p and miR-30a-5p are annotated in orange and purple respectively.

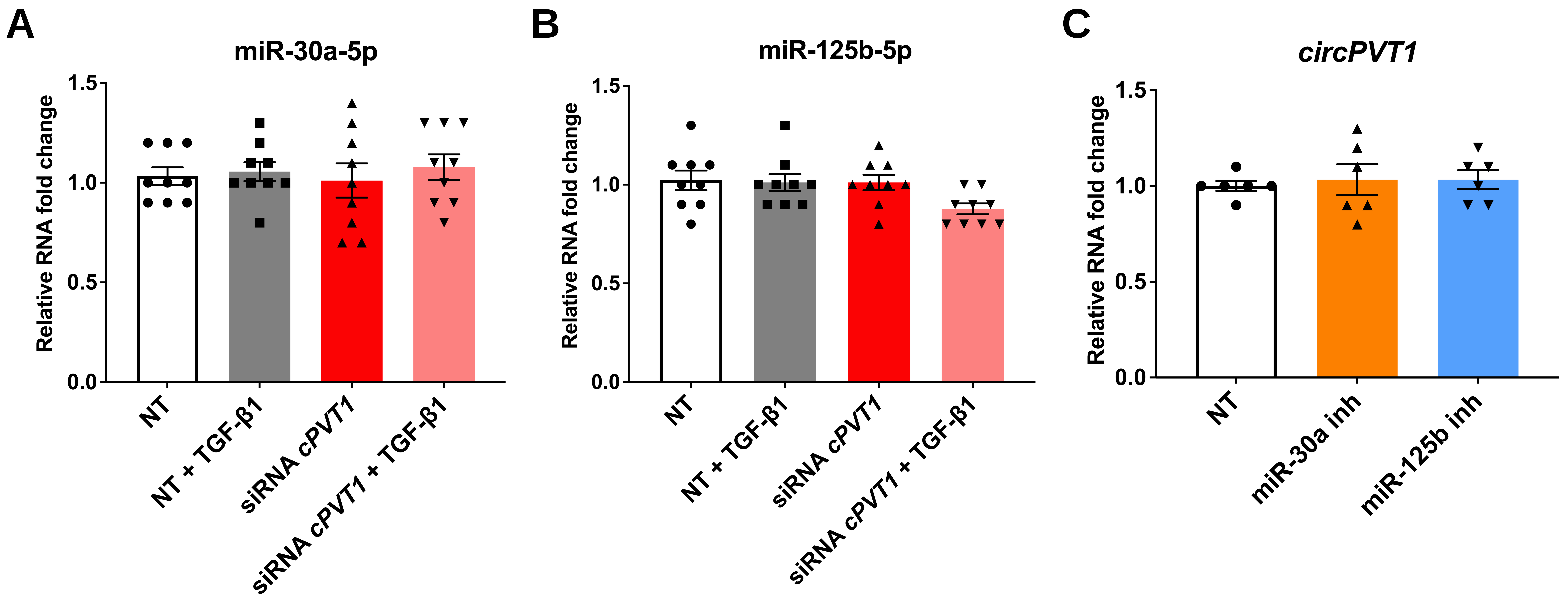

Figure S11. miR-30a-5p, miR-125b-5p, and *circPVT1* levels were not changed upon *circPVT1* or miRNA knockdown. Expression levels of miR-30a-5p **(A)** and miR-125b-5p **(B)** were not significantly modulated in *circPVT1*-knockdown HCF, both untreated or treated with TGF-β1 (n = 9, differences were not statistically significant). **(C)** Expression of *circPVT1* was not affected by the inhibition of miR-30a-5p or miR-125b-5p (n = 6 independent biological replicates, differences were not statistically significant). RNA levels were measured by RT-qPCR and data are presented in fold change as mean ± SEM.
