# Supplementary material for: Circular PVT1 promotes cardiac fibroblast activation interacting with miR-30a-5p and miR-125b-5p
Source: Cell Death Dis. 2025 Apr 21;16(1):325. doi: 10.1038/s41419-025-07652-7 (PMC12012019; doi:10.1038/s41419-025-07652-7)
Supplement: Supplementary file 7 — Original western blots [file 41419_2025_7652_MOESM7_ESM.pdf]

Original western blots

A

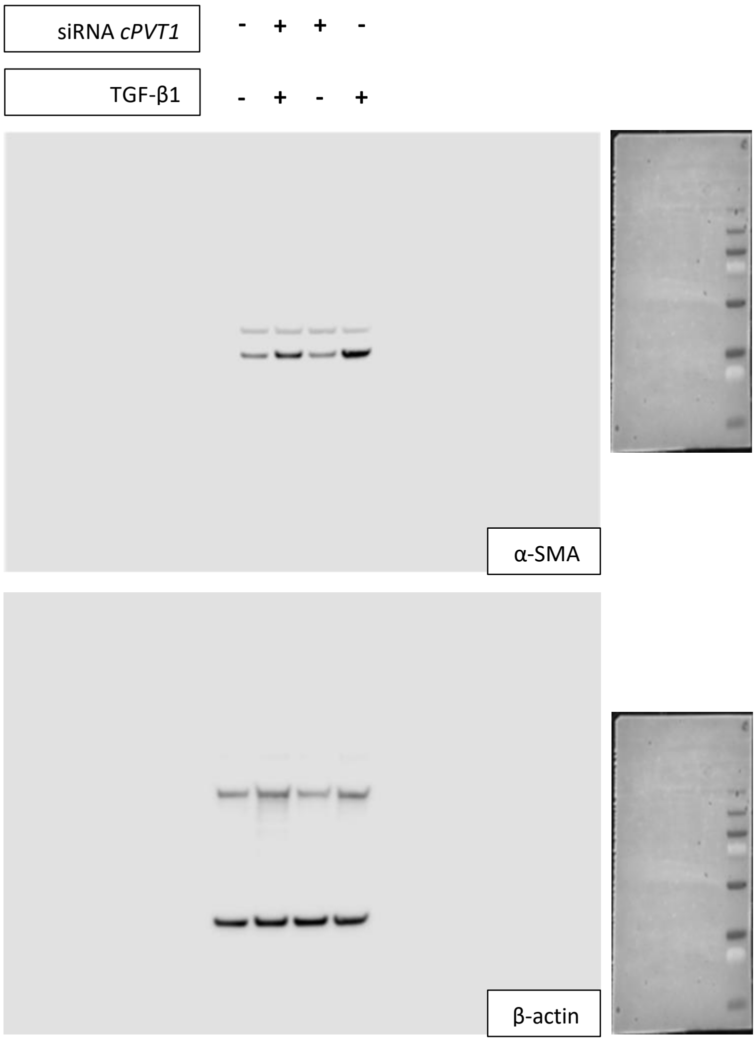

B

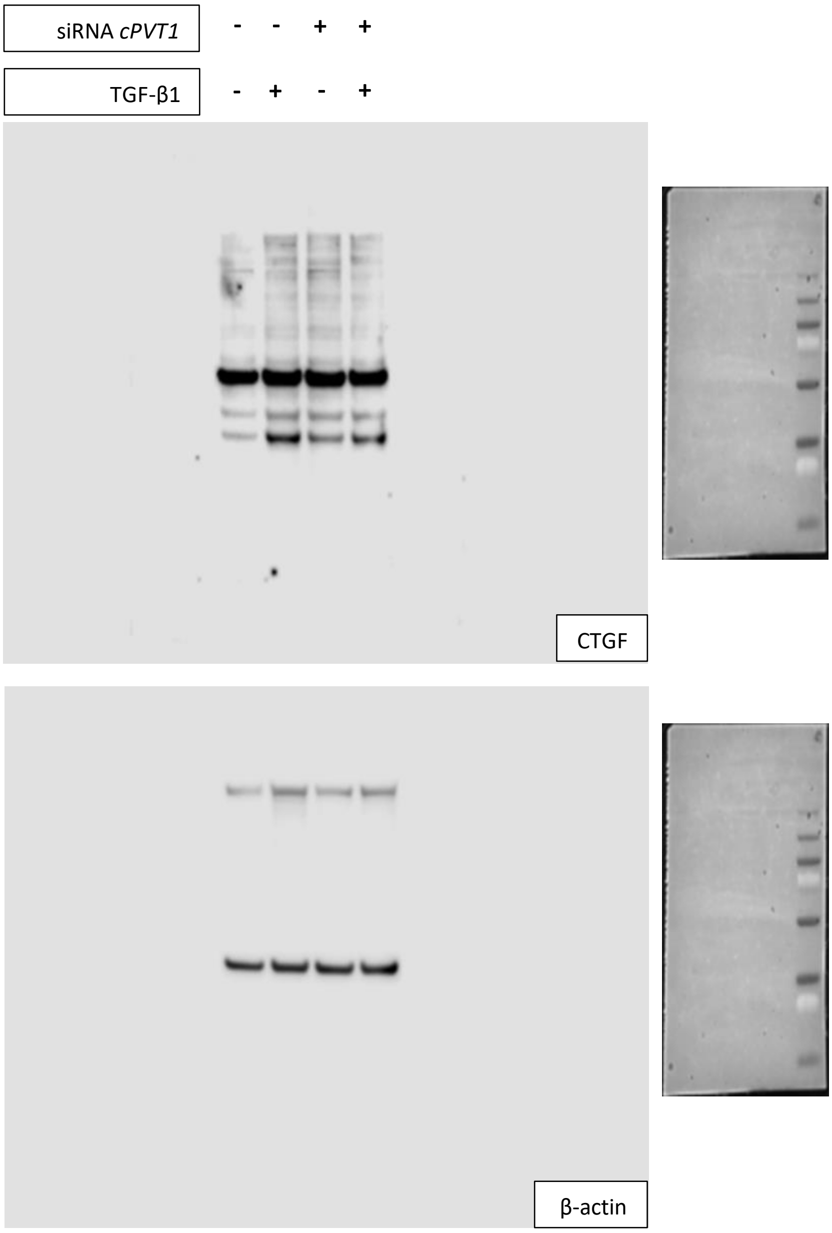

C

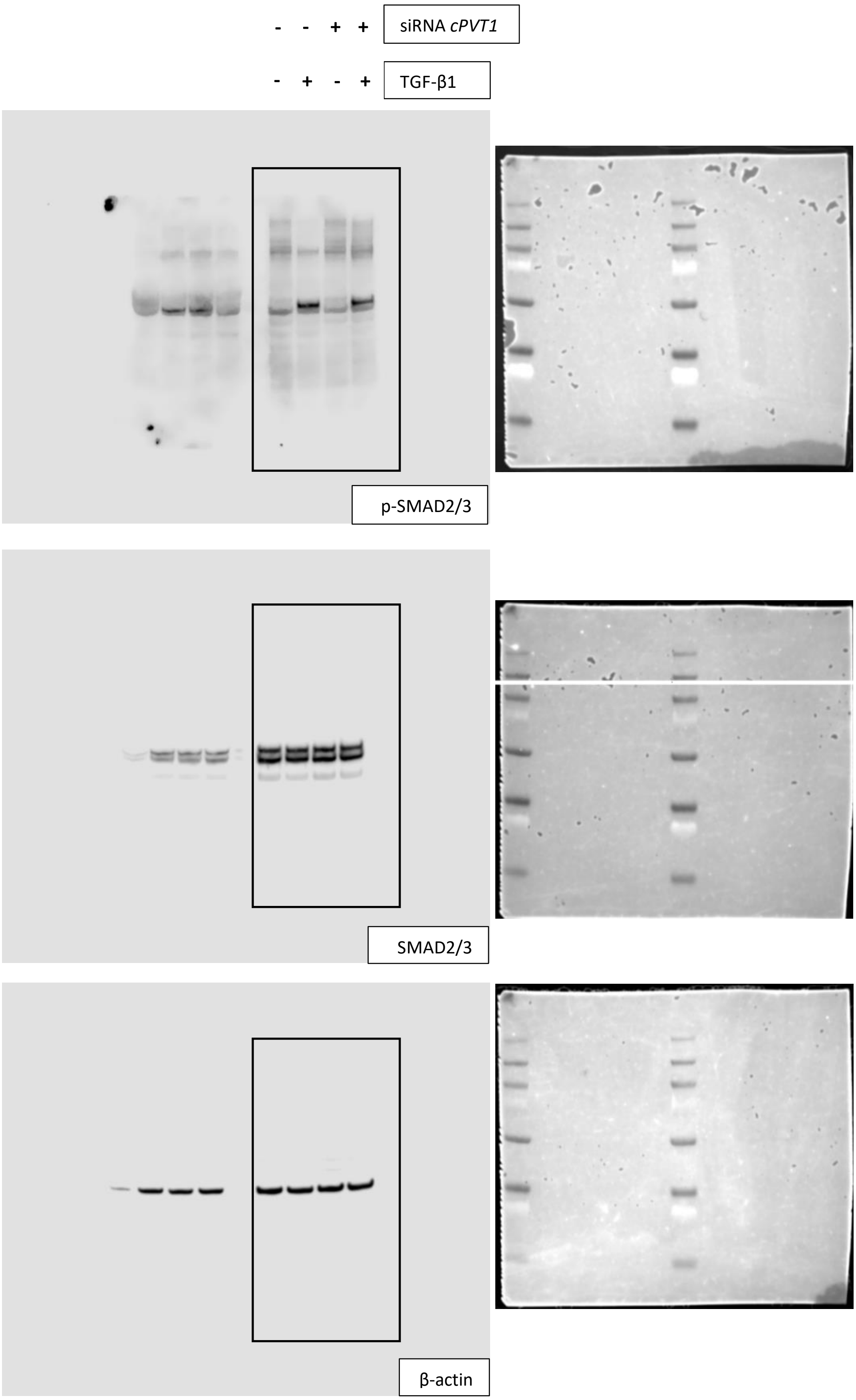

Full length uncropped original western blots. (A) Representative western blot of α-SMA protein level in HCF transfected with non-targeting siRNA or *circPVT1*_1 siRNA (siRNA *cPVT1*) and then stimulated with TGF-β1 for 24h (relative to Figure 2D). **(B)** Representative western blot of CTGF protein level in HCF transfected with non-targeting siRNA or *circPVT1*_1 siRNA (siRNA *cPVT1*) and then stimulated with TGF-β1 for 24h (relative to Figure 2E). **(C)** Representative western blot of p-SMAD2/3 and total SMAD2/3 protein levels in HCF transfected with non-targeting siRNA or *circPVT1*_1 siRNA (siRNA *cPVT1*) and then stimulated with TGF-β1 for 1h (relative to Figure 3). β-actin was used as loading control; a 11-250 kDa protein ladder was used as molecular weight reference.
